# Hybrid material based on hyaluronan hydrogels and poly(l-lactide*-co-*1,3-trimethylene carbonate) scaffolds toward a cell-instructive microenvironment with long-term *in vivo* degradability

**DOI:** 10.1016/j.mtbio.2022.100483

**Published:** 2022-11-01

**Authors:** Tove Kivijärvi, Øyvind Goksøyr, Mohammed A. Yassin, Shubham Jain, Shuntaro Yamada, Alvaro Morales-López, Kamal Mustafa, Anna Finne-Wistrand

**Affiliations:** a Department of Fibre and Polymer Technology, KTH Royal Institute of Technology, Stockholm, Sweden; b Centre of Translational Oral Research (TOR), Department of Clinical Dentistry, University of Bergen, Bergen, Norway; c Department of Oral and Maxillofacial Surgery, Haukeland University Hospital, Bergen, Norway

**Keywords:** Polyester scaffold, Hyaluronan hydrogel, *In vivo* degradation, 3D printing, Salt-particulate leaching

## Abstract

Degradable polyester-based scaffolds are ideal for tissue engineering applications where long-term structural integrity and mechanical support are a requisite. However, their hydrophobic and unfunctionalized surfaces restrain their tissue-mimetic quality. Instead, hyaluronan (HA) hydrogels are able to act as cell-instructive materials with the ability to recapitulate native tissue, although HA is rapidly metabolized *in vivo*. Taking advantage of these distinctly diverse material properties, a degradable and concurrent hybrid hydrogel material was developed that combines the short-term tissue-relevant properties of bio-orthogonal crosslinked HA with the long-term structural and mechanical support of poly(l-lactide-*co*-trimethylene carbonate) (PLATMC) scaffolds. This method rendered the formulation of transparent, minimally swelling hydrogel compartments with a desirable cell-instructive “local” elastic modulus within the scaffold matrix without impeding key material properties of PLATMC. Long-term degradability over 180 days *in vivo* was realized by the integral PLATMC scaffold architecture obtained through either extrusion-based 3D printing or salt-particulate leaching. Intrinsic diffusion capacity within the hydrogel elicited unaffected degradation kinetics of PLATMC *in vivo*, despite its autocatalytic bulk degradation characteristics displayed when 3D-printed. The effect of the processing method on the material properties of PLATMC markedly extends to its *in vivo* degradation characteristics, and essential uniform degradation behavior can be advanced using salt-particulate leaching. Regardless of the scaffold fabrication method, the polymer exhibited a soft and flexible nature throughout the degradation period, governed by the rubbery state of the polymer. Our results demonstrate that the physicochemical properties of the hybrid hydrogel scaffold endow it with the potential to act as a cell instructive microenvironment while not affecting key material properties of PLATMC postprocessing. Importantly, the HA hydrogel does not adversely impact the degradation behavior of PLATMC, a vital aspect in the fabrication of tissue engineering constructs. The results presented herein open new avenues for the adoption of concurrent and well-defined tissue-relevant materials exhibiting the potential to recreate microenvironments for cell encapsulation and drug delivery *in vivo* while providing essential structural integrity and long-term degradability.

## Highlights

• Concurrent cell-instructive hyaluronan hydrogel and structurally defined polyester scaffold.
• Hybrid material realized without impeding key material properties of the polymers.
• Transparent and minimally swelling hydrogel compartments exhibited “local” elastic modulus.
• Polyester scaffolds enabled long-term degradability over 180 days *in vivo*.
• Scaffold processing method have an instrumental effect on the *in vivo* degradation behavior of the polymer.

## Graphical abstract

**Figure:**
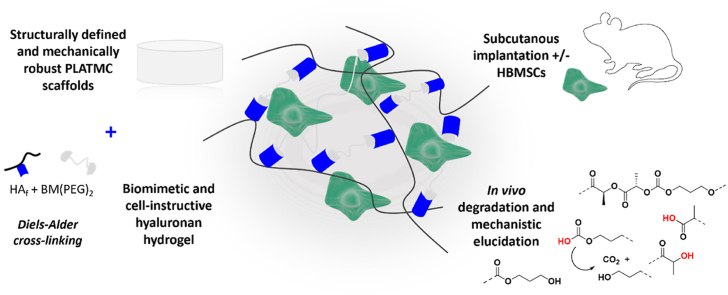

## 1 Introduction

Degradable polyester-based scaffolds have been used extensively as tissue engineering constructs, due to their favorable mechanical strength, diverse processability options available, and tailorable degradation behavior [1,2]. Importantly, these types of scaffolds provide mechanical support and structural integrity for the progression of new tissue formation, while being degraded once the formed tissue requires less support. Although a promising type of material, this class of polymers is relatively hydrophobic and lacks inherent cell-interaction abilities. To circumvent this problem, functionalization of monomers, polymers or postprocessing modifications is used [[3], [4], [5]]. Although their synthetic steps are sometimes tedious, these methods ultimately affect the final properties of the polyester-based scaffolds. During postprocessing treatments, the surface is eroded, which may not only yield more cell-instructive scaffolds but also affect the scaffolds' mechanical properties, polymer crystallinity, and ultimately, but often overlooked, its degradation behavior [6,7]. With the advent of today's processing methods approaching a stage where predictable and defined polyester-based scaffolds can be fabricated in a reproducible manner, functionalization methods that circumvent changes to the physicochemical properties of the polyesters, are needed.

Hydrogels are water-swollen polymer networks with properties resembling the tissue-like elasticity of the extracellular matrix. They are ideal materials for recreating cell-instructive microenvironments, enabling essential diffusion capacity while providing cell encapsulation abilities. Compared to polyester-based scaffolds, the elastic nature of hydrogels allows for a cell-protective environment while allowing the cells to spread and adopt more freely in three dimensions. Hyaluronan (HA)-based hydrogels have received particular attention due to the abundance of HA in all human tissue types [8]. It is hydrophilic and involved in the mediation of cell behavior, such as adhesion and migration, as well as binding to specific cell-surface receptors such as CD44 and RHAMM [9]. Depending on its molar mass, it can also act as an anti-inflammatory substance and promote wound repair. Cell delivery using tissue-relevant HA hydrogels has been shown to be a promising strategy, resulting in high cell affinity, cell seeding efficacy and viability after transplantation [10]. Although a powerful cell delivery platform, native HA is rapidly metabolized in the human body within a day up to 2–3 weeks depending on the tissue type [9]. Even though the crosslinking of HA leads to its prolonged presence, the degradation of HA in combination with extensive swelling of the hydrogel eventually leads to high water intake and dissolution. For tissue repair where regeneration takes place over a few months, the structural integrity of the three-dimensional matrix over a longer period is vital.

Polyester-based scaffolds and hydrogels are complimentary materials used in many ways for tissue engineering applications. Therefore, it is not unsurprising that hybrid structures combining the favorable properties of each individual material have been developed in recent years**.** [[11], [12], [13], [14], [15]] We therefore envisaged to develop a hybrid material combining the short-term tissue relevant properties of HA hydrogels with the long-term mechanical support and structural integrity offered by PLATMC-based scaffolds.

The processability of PLATMC offers a wide range of structural freedom, and porous scaffolds can, for example, be produced through particulate leaching [[16], [17], [18]] or direct extrusion-based 3D printing [19]. Salt-particulate leaching is a conventionally used method offering the advantage of fabricating scaffolds with high porosity without affecting the molar mass of the polymer. A limitation of using this method is that it results in an uncontrolled pore forming process, thereby decreasing its reproducibility in terms of scaffold architecture. Additive manufacturing has emerged as a leading way of fabricating scaffolds with high resolution due to its architectural and geometrical freedom enabling tailorable strand size and thickness with high spatial control. Among these, extrusion-based 3D printing has been extensively explored, although a major drawback of this method is the elevated temperature necessary to melt the polymers that can lead to thermal degradation. We have previously described how extrusion-based 3D printing affects both the molar mass and crystallinity of the polymer [19], although how these altered properties affect the degradation behavior of PLATMC remains unanswered.

Understanding the polymer degradation behavior is crucial when fabricating scaffolds for tissue engineering purposes [1,6,20,21]. The scaffold needs to provide sufficient structural and mechanical integrity during tissue formation but must also degrade during the process to leave room for new tissue to foster. Therefore, a cornerstone in the development of successful tissue engineering materials is their degradation properties. *In vitro* degradation studies are typically used to provide insight into the *in vivo* degradation behavior. However, this does not recapitulate the complexity of the environmental factors to which the material will be exposed and may lead to different outcomes between *in vitro* and *in vivo* degradation [22]. While effort has been made to develop new tools for real-time assessment of degradation rates [23], ultimately, the clinical outcome of a material is dictated by its response *in vivo* [21]. A major focus within the scientific community has been on understanding the *in vitro* degradation behavior of PLATMC [16,[24], [25], [26]] while the *in vivo* degradation process remains less explored [27,28]. The *in vivo* degradation behavior of PLATMC has been determined for films [27], drug release carriers [29], and recently for cardiac occluders [28], but the degradation behavior of scaffolds fabricated from semicrystalline PLATMC has not yet been studied.

**Fig. 1:**
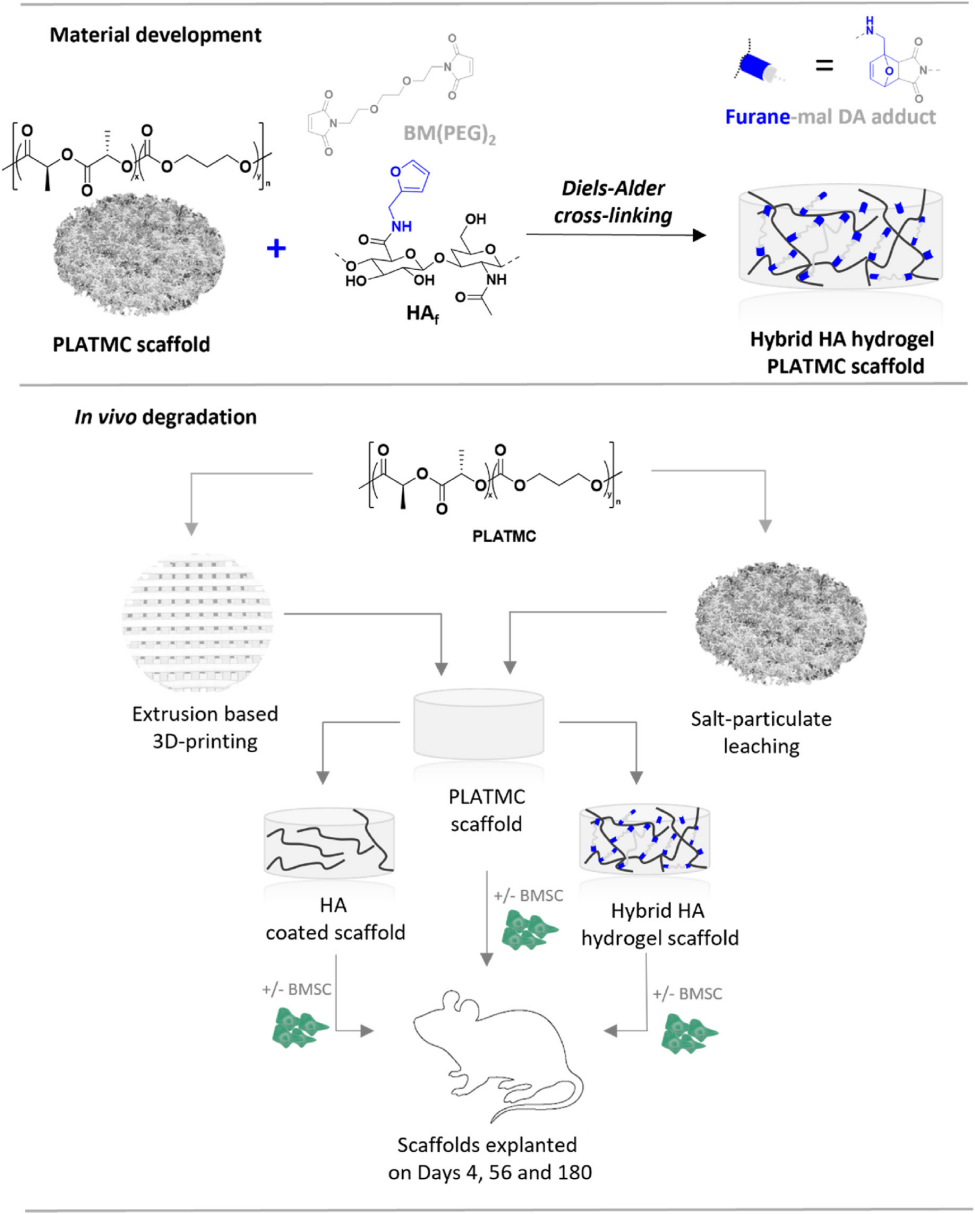
Development of hybrid hydrogel scaffolds based on bioorthogonally crosslinked furan-modified hyaluronan (HA_f_) and 1,8-bismaleimido-diethyleneglycol (BM(PEG)_2_) together with poly(l-lactide-*co*-trimethylene carbonate) (PLATMC) scaffolds. Medical-grade PLATMC was then processed through 3D printing or salt-leaching and hybrid HA hydrogel scaffolds formulated before being subcutaneously implanted into rats for evaluation of their *in vivo* degradation behavior with or without the addition of bone marrow derived stem cells (BMSC). As control groups, native PLATMC scaffolds or PLATMC scaffolds coated with hyaluronan (HA) were used.

The aim of the current study was to enhance the cell-mediating properties of PLATMC-based scaffolds without affecting key material properties such as molar mass, crystallinity, and, especially, its degradability. Our rationale was that a hybrid system based on HA hydrogel and PLATMC scaffolds would concurrently endow short-term cell instructive properties with long-term structural and mechanical support (Fig. 1). Our hypothesis was that the favorable physicochemical properties of HA would allow for sufficient diffusion capacity within the hybrid hydrogel scaffold so that the degradation characteristics of PLATMC would not be altered. Guided by the importance of understanding material degradation behavior in response to the tissue microenvironment, we centered on the *in vivo* degradation behavior of the hybrid hydrogel scaffolds. To illustrate its wide utility, we processed medical grade PLATMC through two different conventionally used scaffold fabrication methods: salt-particulate leaching and extrusion-based 3D printing. Hybrid HA hydrogel scaffolds were thoroughly developed and characterized for their potential as a cell-instructive microenvironment. To elucidate the degradation characteristics of PLATMC, the hybrid HA hydrogel scaffolds were subcutaneously implanted into tissue pockets on the backs of rats and explanted after 4, 56, and 180 days to evaluate their key material properties.

## 2 Results & discussion

### 2.1 Development of a hybrid hydrogel scaffold

**Fig. 2:**
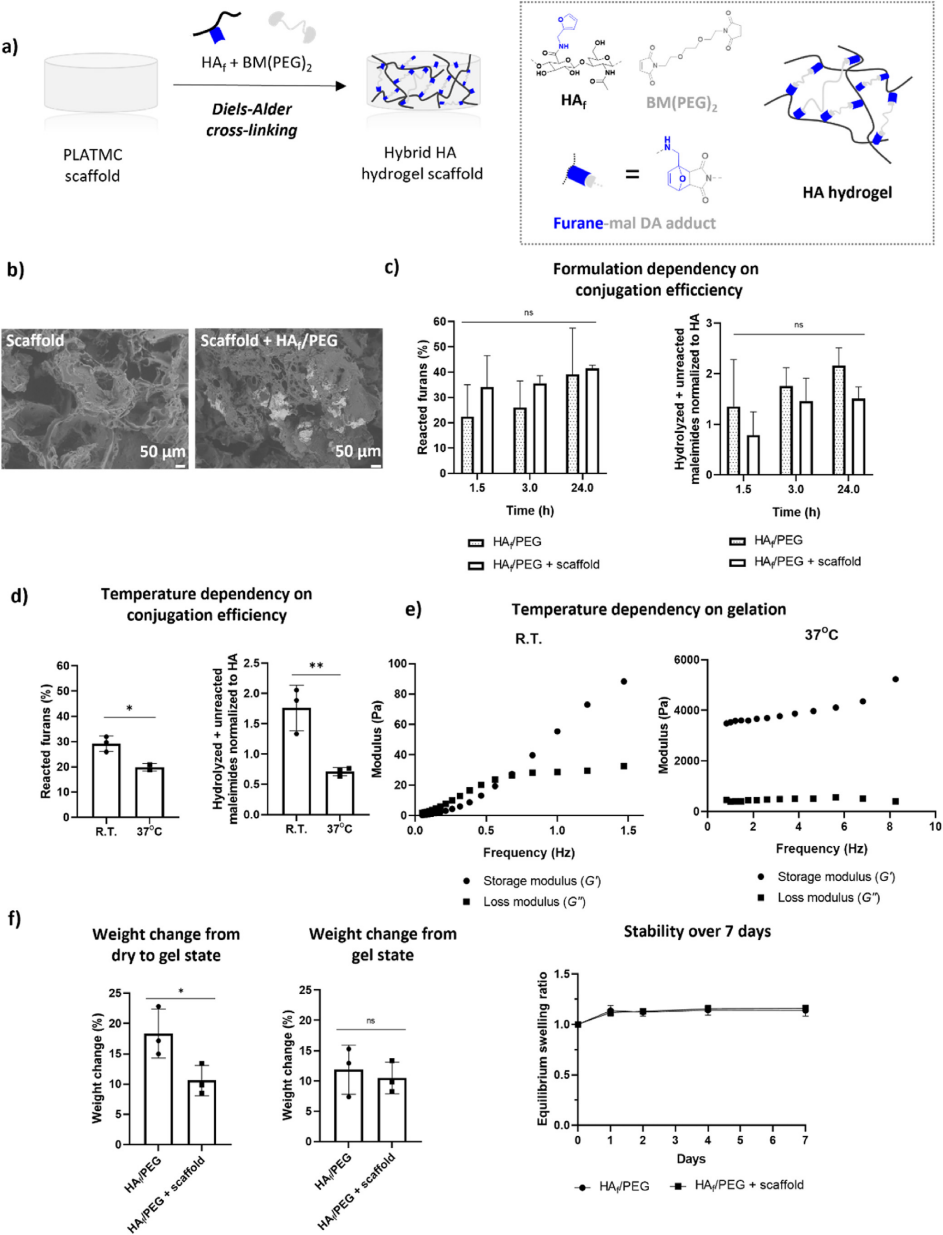
Development and final physicochemical properties of the hybrid HA hydrogel PLATMC scaffold. a) Schematic representation of the Diels-Alder crosslinking of HA_f_ and BM(PEG)_2_ within PLATMC scaffolds; b) representative SEM images of PLATMC scaffolds without (left) and with 5.0% w/v HA_f_/PEG gel ​+ ​scaffold (right). Images were taken after freeze-drying and from top surface with ×200 magnification; c) conjugation efficiency over time comparing gels with and without a scaffold at R.T. using 5.0% w/v Ha_f_ and BM(PEG)_2_; d) temperature dependency on the conjugation efficiency of 0.5% w/v HA_f_ and BM(PEG)_2_ within the scaffolds after 90 ​min; e) representative frequency sweep demonstrating the viscoelastic behavior of HA_f_ ​+ ​BM(PEG)_2_ after preincubation at R.T. or 37 ​°C for 24 ​h, confirming crosslinking at 37 ​°C; and f) swelling/stability study of 5.0% w/v HA_f_/PEG gel or HA_f_/PEG gel ​+ ​scaffold incubated at 37 ​°C in phosphate buffer saline (PBS). Weight increase over initial 48 ​h from the dry state (freeze-dried) or gel point to the equilibrium gel state (left figures) and equilibrium swelling ratio over 7 days (right figure). Minimally swollen and stable transparent gels were formed with <4% variation in mass over 7 days. Statistical significance was determined using a T test: N.S. ​= ​not significant, ∗p ​≤ ​0.05, ∗∗p ​≤ ​0.01, ∗∗∗p ​≤ ​0.001, ∗∗∗∗p ​≤ ​0.0001 (n ​= ​3; mean ​± ​SD).

Click based chemistry, such as the Diels-Alder cycloaddition reaction, is a convenient method for the preparation of hydrogels in the presence of cells, cytokines, growth factors or peptides [30,31]. Diels-Alder cycloaddition reactions between an electron-rich diene and an electron-poor dienophile are completely orthogonal toward ester- and carbonate bonds, an important aspect for gel formation in presence of PLATMC-based scaffolds. Importantly, the reaction can be performed in water-based media without the addition of a catalyst [32], which are both crucial advantages for the preparation of materials for biomedical applications. Motivated by this, we centered the development of hybrid HA hydrogel scaffolds on this type of chemistry (Fig. 2).

#### 2.1.1 Development of a hybrid HA hydrogel PLATMC scaffold using Diels-Alder chemistry

Employing bismaleimide-modified polyethylene glycol (PEG) [33], peptides [[34], [35], [36], [37]], or HA derivatives [38] as dienophiles with furan-modified HA as the diene, Diels-Alder cycloaddition has been previously used to create cell-instructive microenvironments. Taking advantage of this type of chemistry, we envisaged the development of PLATMC scaffolds immersed in Diels-Alder-crosslinked high-molar mass HA hydrogels (Fig. 2a). To explore this possibility, HA (*M*_*n*_ 1400–1800 ​kg ​mol^−1^) was modified with furan moieties to a degree of substitution of 60%, as confirmed by ^1^H Nuclear Magnetic Resonance (NMR) (Fig. S1). Furan-modified HA (HA_f_) was then dissolved in 100 ​mM morpholine ethane sulfonic acid monohydrate (MES) buffer (pH 5.5) and immersed within the salt-leached PLATMC scaffolds. We chose to use salt-leached scaffolds as a model scaffold, as the diffusion capacity is expected to be lower in the salt-leached scaffolds because its gap size is smaller than that of 3D-printed scaffolds. Therefore, we expected this scaffold to be more complex to formulate into a hybrid hydrogel scaffold. The Diels-Alder crosslinking reaction was initiated by the injection of the crosslinker 1,8-bismaleimido-diethyleneglycol (BM(PEG)_2_) (1 equiv. maleimide to furan moiety) and transparent HA hydrogels forming within and around the salt-leached PLATMC scaffolds, i.e., hybrid HA hydrogel scaffolds (Fig. 2a and b and Fig. S6). To quantify the conjugation efficiency, the glycosidic bonds of the HA_f_ main chain were digested by hyaluronidase, and ^1^H NMR was used to evaluate the number of reacted furans as well as the reactivity of the maleimides (Fig. S2). The percentage of reacted furans was estimated using Equation (1), representing the reactivity of furan moieties on HA_f_. Since the crosslinking efficiency is dictated by both maleimide groups on BM(PEG)_2_ efficiently reacting with HA_f_ polymer chains, the reactivity of the maleimides was also evaluated. Thus, the ratio of hydrolyzed and unreacted maleimides normalized to HA_f_ was estimated based on Equation (2), presented as an inverse measurement of the maleimide reactivity. Initially, the conjugation efficiency over time was compared between the HA_f_/PEG formulation and the hybrid HA_f_/PEG formulation within salt-leached PLATMC scaffolds (Fig. 2c and Fig. S3a). The reaction was allowed to take place at room temperature (R.T.) using a final concentration of 5.0% w/v, and the conjugation efficiency was evaluated after 90, 180 and 1440 ​min. No significant differences were shown over time, and both formulations achieved ≈40% reacted furans within 24 ​h of incubation. Similar maleimide reactivity was shown in both cases, with a slightly lower extent of hydrolysis or unreacted maleimides in the case for the formulation of hybrid HA_f_/PEG within the salt-leached PLATMC scaffolds.

#### 2.1.2 Temperature-dependency on the conjugation efficiency of the crosslinking reaction

The reaction temperature was also varied to evaluate its dependency on the conjugation efficiency (Fig. 2d and Fig. S3b). The number of reacted furans was significantly higher when the reaction was allowed to take place at R.T. than the reaction at 37 ​°C, corresponding to 36 ​± ​2.9% reacted furans at R.T. and 20 ​± ​1.5% reacted furans at 37 ​°C after 90 ​min (out of 60% being feasible). However, the amount of hydrolyzed and unreacted maleimides was also significantly higher at R.T., suggesting that even though the furans had reacted to a higher extent at R.T. than at 37 ​°C, the reactivity of the crosslinker BM(PEG)_2_ was lower due to the higher degrees of hydrolyzed and unreacted maleimides. To confirm this hypothesis, rheological measurements were carried out on the HA_f_/PEG formulations after 24 ​h but without salt-leached PLATMC scaffolds (Fig. 2e). Upon crosslinking, both the storage modulus (G') and loss modulus (G'′) are expected to increase due to the higher molar mass and structural order obtained upon network formation, giving rise to higher molecular motion frequencies [39]. The ratio between G'′ and G' corresponds to tan- *δ*, and when these values are lower than 1, the material exhibits an elastic nature (Fig. S4). Therefore, preformed hydrogels exposed to the oscillation frequency are expected to have higher G' values than G'′ values. Instead, a viscous liquid is expected to have higher G'′ values than G' values, and if a crossover frequency point is observed where G' ​= ​G'′ (gel point), under the applied rheological conditions, the frequency induces enough yield stress so that the material becomes predominantly elastic and therefore appears as a physical network. As expected, frequency sweeps from 0.1 to 10 ​Hz showed that the formulation made at R.T. had a gel point of approximately 0.6 ​Hz, thereby demonstrating that crosslinking had not occurred prior to the rheological measurements. This supports the results obtained from the ^1^H NMR conjugation efficiency experiments; even though the furans had reacted to a large extent, the hydrolysis of the maleimides prevented enough crosslinking to occur at R.T. for the formulation to form a hydrogel. Conversely, the formulation prepared at 37 ​°C demonstrated a distinctly higher G' than G'′, confirming that crosslinking had taken place at 37 ​°C. The difference in elasticity between the formulations made at R.T. and 37 ​°C (≈100 ​Pa compared to ≈4000 ​Pa) further supports their difference in ordered structure. Elasticity represents the ability of a gel to resist alterations in shape by an applied force, meaning that the hydrogel made at 37 ​°C (with a higher G') better resisted deformations than the formulation made at R.T. (with a lower G'). This is because longer polymer chains imply longer relaxation times, and therefore, low or noncrosslinked networks (less ordered) give rise to lower molecular motion frequencies.

#### 2.1.3 Viscoelastic behavior of the Diels-Alder crosslinked hydrogel

Matching the physicochemical properties of hydrogels and scaffolds with the inherent properties of the desirable tissue environment is vital. Although crosslinked HA-based hydrogels have emerged as a tissue-relevant approach to create microenvironments, a limiting factor is the different mechanical properties of hydrogels compared to tissue types other than soft tissue [40]. Solid tissues are exposed to various mechanical loads, which makes the elastic properties integral for their function. To improve the mechanical properties of hydrogels, the introduction of ceramics [41] or fibrous components [42] into HA hydrogels has been shown to be beneficial [13]. Mechanical properties of the extracellular matrix also impact gene expression through mechanotranduction [43], and the modulus of the microenvironment has been shown to influence stem cell spreading, cell fate, and macrophage polarization [[44], [45], [46]]. Although the modulus of the hybrid HA hydrogel PLATMC scaffold differs, knowledge of the HA hydrogel modulus is important for potential cell encapsulation and delivery strategies, where cells would potentially sense the local modulus from the HA hydrogel. We therefore evaluated the rheological properties of the crosslinked HA hydrogel (i.e., without a scaffold). The storage modulus (*G′*) of the 0.5% w/v HA hydrogel showed a weak frequency dependency at 37 ​°C, while the loss modulus (*G″*) was unchanged over 0.1–10 ​Hz (Fig. 2e). The crosslinked hydrogel exhibited a linear equilibrium modulus plateau with respect to frequency, corresponding to an elastic storage modulus of 3.03 ​± ​0.37 ​kPa for a frequency change of 0–10 ​Hz (Fig. S5). This correlates well with an elastic modulus somewhere between that of the brain and that of muscle tissue, while the modulus of cartilage or collagenous bone is substantially higher (100–1000 ​kPa) [[47], [48], [49]]. Mesenchymal stem cells have shown lineage specificity depending on matrix stiffness, with an elastic modulus of 11 ​kPa leading to preferred osteogenic differentiation unlike a lower modulus leading to adipogenic differentiation [48,50,51]. This demonstrates the importance of providing an integrated PLATMC scaffold within the hydrogel developed for tissue engineering applications where higher substrate elasticity is needed. The compressive modulus of PLATMC scaffolds is between 1 ​kPa and 10 ​MPa depending on fabrication method and experimental conditions used [19,52].

#### 2.1.4 Viscoelastic behavior of the non crosslinked HA formulations

We further compared the rheological measurements of HA_f_/PEG formulated at R.T. with those of furan-modified HA and unmodified HA (Fig. 2e and Fig. S4). The rheological characteristics of these formulations are important, as upon *in vivo* degradation, the hydrogel will likely approach a viscoelastic behavior closer to that of semi-crosslinked HA_f_/PEG, HA_f_ and HA. The gel point of HA_f_/PEG formulated at R.T. was significantly higher in terms of modulus (27.8 ​± ​1.69 ​Pa) than that of furan-modified HA (7.9 ​± ​2.08 ​Pa) and that of unmodified HA (5.5 ​± ​0.31 ​Pa). These results demonstrate that with a sufficient applied oscillation frequency, all HA formulations appeared as elastic solids due to the restricted movements of polymer chains under the rheological conditions applied and likely due to the high-molecular weight HA used [39]. This behavior is governed by two properties. Initially, upon an applied force, the viscoelastic response of a polymer network is governed by the intrinsic rate of disentanglement of the polymer chains, whereby the chain network can release its yield stress. However, above G' ​= ​G'′ and at high frequencies, long polymer chains will fail to rearrange themselves within the timescale of the imposed mechanical motion, resulting in a stiffening of the material so that it appears as a temporary elastic solid. The gel point of HA_f_/PEG formulated at R.T. being significantly higher than that of HA_f_ and that of HA suggests that this formulation formed a substantially stronger physical network, likely due to chemical crosslinks occurring during the rheological measurements conducted at 37 ​°C.

#### 2.1.5 Diffusion capacity of the Diels-Alder crosslinked HA hydrogel

The diffusion capacity of a material is instrumental in controlling the delivery and activity of nutrition within the matrix as well as preventing the accumulation of unwanted components. This is especially important for polyester-based scaffolds that release acidic byproducts and are typically bulk-degrading. Therefore, we used rheological measurements to elucidate the porosity characteristics of the HA hydrogel. The apparent average mesh size (*ξ*_*a*_) of the HA hydrogel was approximated using the elastic storage modulus of 3.03 ​± ​0.37 ​kPa obtained in the frequency range of 0–10 ​Hz and Equation (3), [53]. The mesh size, i.e., the distance between two entanglement points, was estimated to be 11.26 ​± ​0.42 ​nm and can be considered a crude estimation of the gel porosity; however, in reality, swelling as well as macroscopic inhomogeneities present in the network will increase the effective mesh size. The mesh size estimated for the developed HA hydrogel is consistent with the mesh size of other hydrogels used as biomaterials shown to promote the diffusion of nutrients, small-molecule drugs and growth factors while preventing nonspecific accumulation of macromolecules within the netowrk [53].

#### 2.1.6 Swelling and stability properties of the HA hydrogel PLATMC scaffold

Finally, we assessed the swelling and stability properties of the HA hydrogel and the hybrid HA hydrogel salt-leached PLATMC scaffold (Fig. 2f). The samples were incubated with PBS at 37 ​°C from either the dry state (after freeze-drying) or the gel state (gel point/preformed gel). The weight of scaffolds alone were 17.8 ​± ​3.2 ​mg, and 44.4 ​± ​4.7 ​mg after formulation of hybrid HA hydrogel scaffold following freeze-drying. The weight increase (Equation (4)) of the HA hydrogel from dry state to the gel state over 24 ​h was 18.3 ​± ​4.05%, while the weight increase of the hybrid HA hydrogel scaffolds was 10.6 ​± ​2.51%, illustrating a significantly smaller swelling capacity when the salt-leached PLATMC scaffolds were included in the gel (Fig. 2f). The stabilities of the HA hydrogel and hybrid HA hydrogel salt-leached PLATMC scaffolds were also evaluated over 7 days in PBS using the equilibrium swelling ratio (Equation (5)). Over the first day, the weight increase of the HA hydrogel was 11.9 ​± ​4.06%, while the weight increase of the hybrid HA hydrogel scaffold was 10.5 ​± ​2.62% during the same time period *(i*.*e.*, equilibrium swelling). Between Day 1 and Day 7, <2% of the weight change was observed for the HA hydrogel, and <4% weight change was observed for the hybrid HA hydrogel scaffolds. These results demonstrate that the formulated hydrogels remained unaffected over 7 days, consistent with minimal swelling behavior. The high stability of hydrogels represents an advantage over extensive swelling, as network degradation in combination with extensive swelling leads to high water intake and, eventually, dissolution of the polymer network.

### 2.2 In vivo degradation of PLATMC

**Fig. 3:**
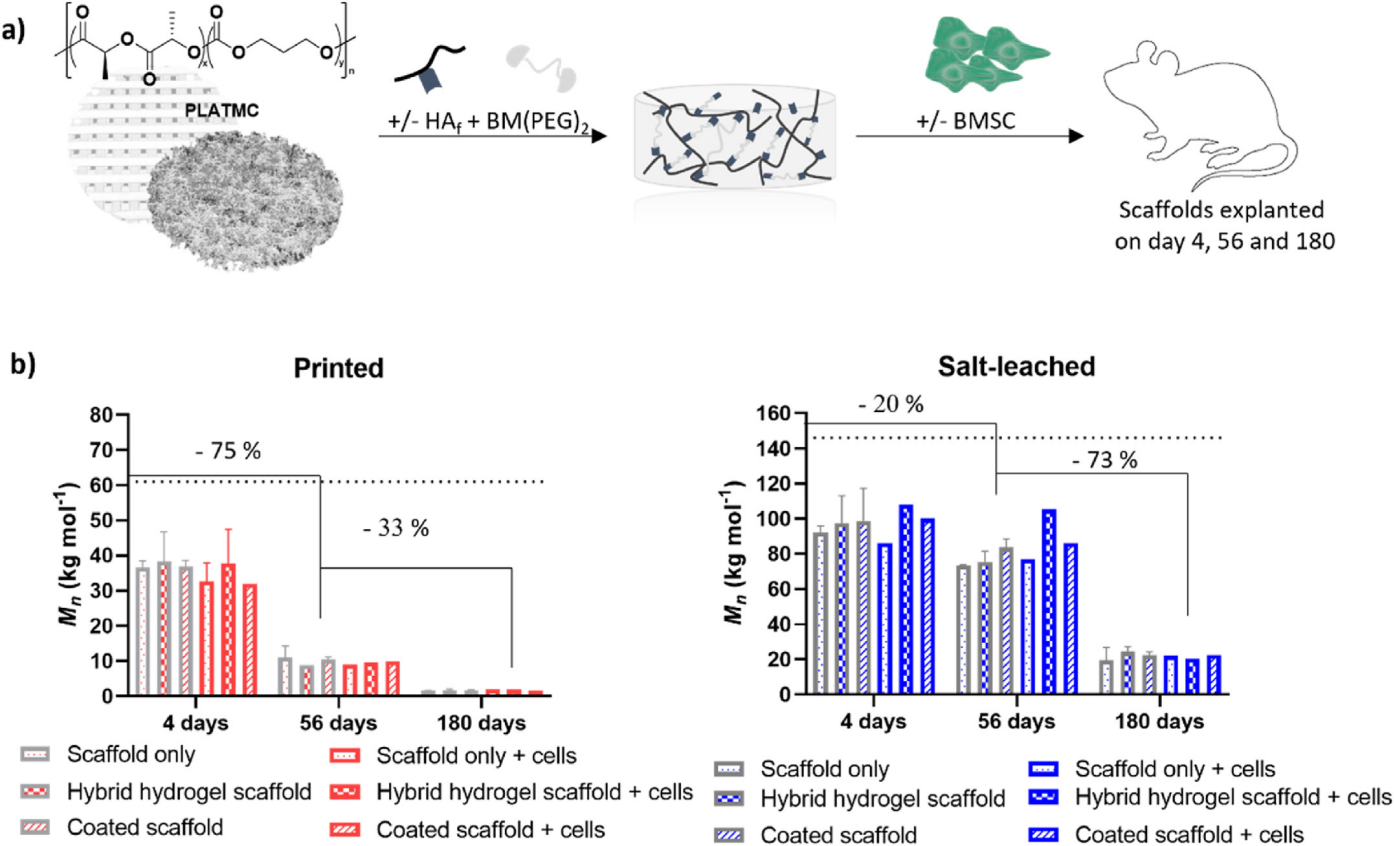
Changes in the molar mass of PLATMC during the *in vivo* degradation period. Scaffolds are grouped into their respective processing methods: extrusion-based 3D printing (printed or P) and salt-particulate leaching (salt-leached or S). Values were obtained from GPC after explantation of the scaffolds on Days 4, 56, and 180 (n ​= ​2). a) Schematic representation of fabrication of hybrid HA hydrogel scaffolds either using salt-leached or 3D printed scaffolds (hybrid hydrogel scaffold), and subsequently *in vivo* degradation evaluation following implantation with or without cells. Native scaffolds (scaffold only) and HA coated scaffolds (coated scaffold) were also used as controls. b) Evolution of the average molar mass distribution (*M*_*n*_). Values refer to the major peak when the GPC trace displayed a bimodal curve. The dashed line represents the initial *M*_*n*_ after fabrication of the scaffolds.

With the final physicochemical properties of the hybrid HA hydrogel PLATMC scaffold in hand, we turned our attention toward the degradation behavior of the polymer. The hydrolytic degradation of PLATMC is dependent on several factors concerning the material, such as its molar mass, polymer composition, crystallinity, porosity and material processing [21,25]. The complexity of material degradation behavior is further magnified by the environmental surroundings [21]. Molecular chain scission of PLATMC can occur passively through hydrolysis of the ester bonds or actively by enzyme-mediated hydrolysis of primarily the carbonate bonds [54]. The byproducts formed from hydrolytic cleavage of the ester bonds are acidic, potentially affecting the degradation rate through autocatalysis when diffusion of these species is limited [16,24,55,56]. The difference in porosity and porous architecture of salt-leached and printed scaffolds vary substantially [19,52] and the incorporation of HA hydrogels into the scaffolds can potentially affect diffusion within the scaffolds and thereby alter the resorption and degradation behavior of the PLATMC copolymers. To ensure that the HA hydrogel would not adversely affect the degradation behavior of PLATMC, we evaluated and characterized key material properties over 180 days *in vivo* (Fig. 3a).

#### 2.2.1 Fabrication of hybrid HA hydrogel PLATMC scaffolds for *in vivo* degradation assessment

Medical-grade PLATMC was used to ensure that a minimum amount of impurities, such as residual catalysts or monomers, were present in the polymer batch prior to processing, which could affect the degradation kinetics [55]. The polymer was processed through salt-particulate leaching or extrusion-based 3D printing [19,52] resulting in circular scaffolds with a diameter of 8 ​mm and a height of 1–2 ​mm. The porosity of the 3D-printed scaffold was ≈49%, while salt-leached scaffolds had interconnected pores with ≈92% porosity [19,52]^,^ although the gaps within the 3D-printed scaffolds were larger than those of the salt-leached scaffolds. The resulting *M*_*n*_ values of PLATMC scaffolds postprocessing varied substantially between the fabrication methods: 128 ​kg ​mol^−1^ for the polymer in the salt-leached scaffold and half of that, 62 ​kg ​mol^−1^, for the polymer in the printed scaffolds. Printed and salt-leached PLATMC scaffolds were then fabricated into hybrid HA hydrogel scaffolds according to the developed method of cross-linking at 37 ​°C (referred to as hybrid HA hydrogel scaffolds). The same volume (400 ​μL) and concentration (5.0% w/v) of HA_f_ and BM(PEG)_2_ was used regardless of scaffold fabrication method, to adequately compare them. To account for any potential effect of HA on the *in vivo* degradation of PLATMC, scaffolds coated with native HA were also fabricated as a control group (referred to as HA coated scaffolds). Non-functionalized PLATMC scaffolds were also included as a control (referred to as native scaffolds). The scaffolds were freeze-dried and sterilized prior to use (Fig. S6). Each scaffold type was then either preseeded with bone marrow derived stem cells (BMSCs) or used without being cell-loaded. Scaffolds were subcutaneously implanted into tissue pockets on the flanks of rats, an animal model that is typically used to assess the *in vivo* degradation of polyester-based materials [21]. The subcutaneous tissue is composed of loose connective tissue as well as adipose tissue with permeating blood capillaries and perfusing interstitial fluid [57]. The diffusion abilities within the subcutaneous tissue have been compared to those of hydrogels [58]. Therefore, implanting the hybrid hydrogel scaffold subcutaneously allowed the material to be in direct contact with the extracellular matrix, while we expected some form of water diffusion capacity to exist. Explantation of the scaffolds was performed on Days 4, 56, and 180, and key material properties, such as changes in average molar mass (*M*_*n*_ and *M*_*w*_), were analyzed by Gel Permeation Chromatography (GPC). Microstructural analysis was performed by ^1^H and ^13^C NMR, morphology and structure was evaluated by Scanning Electron Microscope (SEM), while thermal properties were evaluated by Differential Scanning Calorimetry (DSC) and Thermal Gravimetric Analysis (TGA).

#### 2.2.2 Effect of the HA hydrogel on the changes in the molar mass distribution of PLATMC during the *in vivo* degradation period

To understand the effect of HA hydrogels on the degradation behavior of PLATMC, we compared the differences in molar mass (*M*_*n*_ and *M*_*w*_) between the hybrid HA hydrogel PLATMC scaffolds and the native scaffolds without hydrogels. As expected, the *M*_*n*_ and *M*_*w*_ of PLATMC decreased with increasing *in vivo* degradation time for all scaffolds (Fig. 3b and S7). Importantly, no difference in *M*_*n*_ and *M*_*w*_ was observed between the hybrid HA hydrogel scaffolds and their respective salt-leached or printed native PLATMC scaffolds. The *M*_*n*_ of PLATMC in the printed scaffold decreased from 62 ​kg ​mol^−1^ postprocessing to 34 ​kg ​mol^−1^ on Day 4 for PLATMC in the hybrid HA hydrogel scaffold. This is comparable to the *M*_*n*_ of 36 ​kg ​mol^−1^ for PLATMC in the printed native scaffold without hydrogel. During the same time interval, PLATMC in the salt-leached scaffold decreased from 128 ​kg ​mol^−1^ postprocessing to 97 ​kg ​mol^−1^ for the polymer in the hybrid HA hydrogel scaffold and 92 ​kg ​mol^−1^ for PLATMC in the native scaffold without the hydrogel. Based on the swelling and stability study performed on the hybrid HA hydrogel scaffold, demonstrating stable hydrogels over at least 7 days in PBS, it is reasonable to assume that the hydrogel would reside within the scaffolds until Day 4. The fact that the molar mass did not differ between the hybrid hydrogel scaffolds and the PLATMC scaffolds during this early time interval, despite the large decrease in molar mass, clearly demonstrates that the hydrogel did not prevent diffusion of oligomeric species out from the bulk of the scaffolds that could otherwise have led to enhanced autocatalysis. No differences between the molar masses of PLATMC in the hybrid HA hydrogel scaffold and the printed or salt-leached scaffolds were observed on Days 56 or 180, and no differences were observed compared to the HA coated scaffold used as control group.

The *in vivo* degradation of PLATMC is influenced not only by passive hydrolysis but also by biological processes, such as enzyme-assisted hydrolysis of primarily carbonate bonds as well as active metabolism [54,59]. Since the hybrid HA hydrogel scaffold exhibited ideal properties for cell delivery purposes, our interest was in elucidating the potential influence of cells on degradation. The degradation behaviors of hybrid hydrogel scaffolds preseeded with BMSCs were therefore compared (Fig. 3b). In all cases, we observed similar molar mass changes over time compared to the scaffolds without BMSCs. Taken together, these results demonstrate that the developed hybrid HA hydrogel scaffold does not alter the PLATMC degradation behavior *in vivo* and demonstrates its potential to be used as a cell delivery platform.

#### 2.2.3 Effect of the scaffold fabrication method on the changes in molar mass distribution of PLATMC during the *in vivo* degradation period

**Fig. 4:**
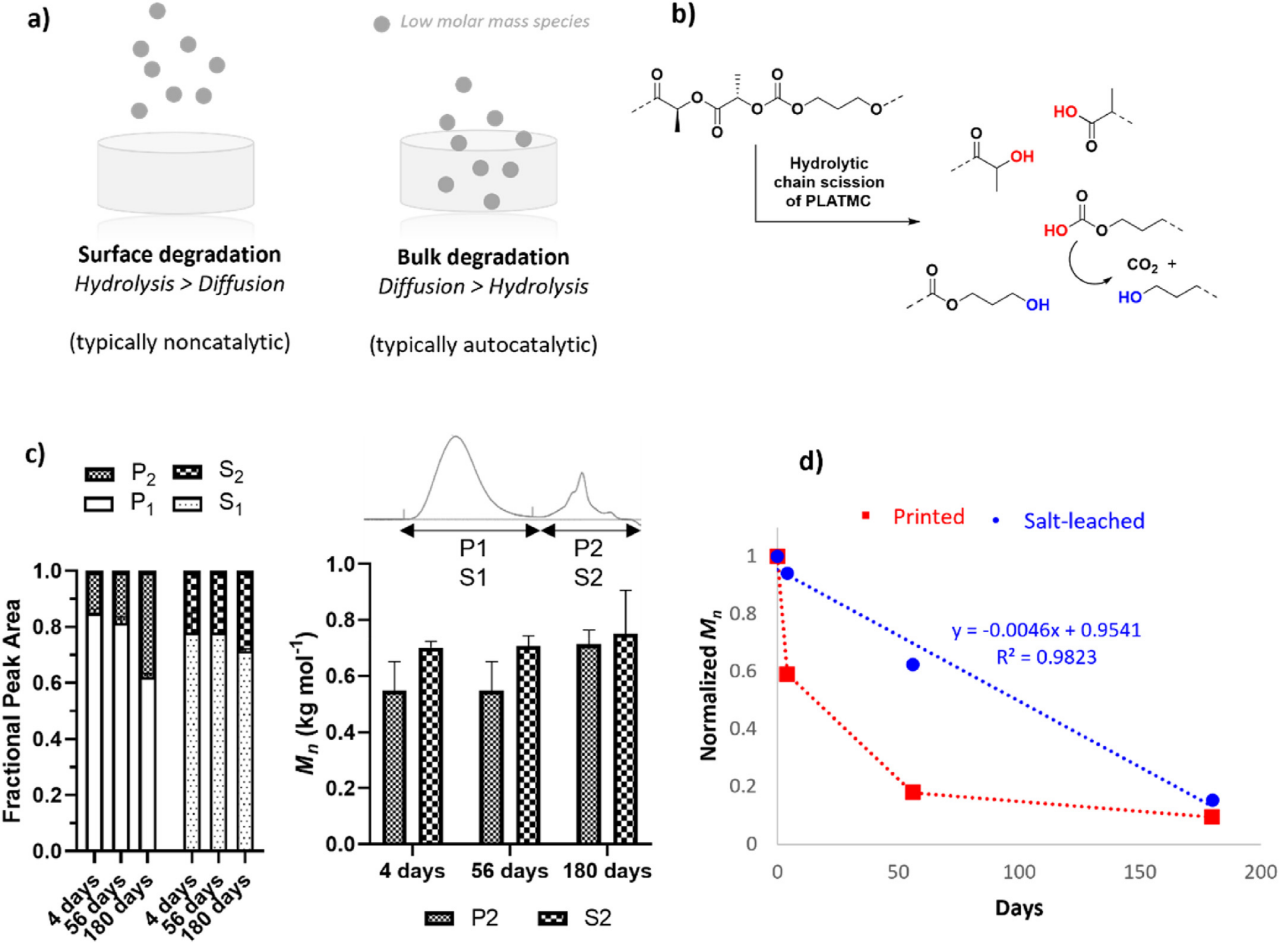
Mechanistic elucidation on the changes in the molar mass of PLATMC during the *in vivo* degradation period. Scaffolds are grouped into their respective processing methods: extrusion-based 3D printing (printed or P) and salt-particulate leaching (salt-leached or S). Values were obtained after explantation of the scaffolds on Days 4, 56, and 180. a) Schematic representation of different erosion modes and degradation mechanisms upon hydrolytic chain scission of PLATMC. b) Representative figure of the byproducts from PLATMC upon hydrolytic chain scission. Red illustrates acidic chain ends. c) Representative figure of the peak area(s) used to determine changes in the molar mass distribution and fractional peak area, where P1/S1 refers to fraction 1 and P2/S2 refers to fraction 2 (small chain fraction) of the bimodal curve in the GPC trace, representing the two individual populations of polymer species. Values were obtained from the native scaffold (scaffold only). d) Representative evolution of molar mass (*M*_*n*_) over the time of the *in vivo* degradation period for PLATMC in extrusion-based 3D printed (bottom/red) and salt-particulate leached (top/blue) scaffolds. *M*_*n*_ was normalized to Day 0 (postprocessing). Values were obtained from the native scaffolds (scaffold only). (For interpretation of the references to color in this figure legend, the reader is referred to the Web version of this article.)

The initial molar mass of polymers has a distinct effect on degradation [55]. This effect is dependent on the type of hydrolytic chain cleavage that occurs (noncatalytic or autocatalytic) and on the modes of erosion (surface or bulk erosion), although these effects are not uncoupled (Fig. 4). The kinetics of chain scission and the kinetics of water diffusion within the material concurrently dictate the degradation rate [6]. Although random, noncatalytic chain scission occurs at the same rate regardless of the starting *M*_*n*_, high-molar-mass polyesters typically degrade faster than low-molar-mass polyesters [60]. This is because polymers with high initial *M*_*n*_ have fewer chains, and therefore, the effect of one chain scission on the molar mass is greater. When autocatalysis is prevalent, this kinetic relationship is offset, and the opposite relationship occurs [55]. The large difference in the *M*_*n*_ values of the printed and salt-leached PLATMC scaffolds encouraged us to elucidate any differences in degradation behavior between the two different scaffold types. During the *in vivo* degradation period, *M*_*n*_ decreased by ​~ ​91% for PLATMC in the printed scaffolds and by ​~ ​85% in the salt-leached scaffolds. While the overall percentage decreases in *M*_*n*_ were similar for the copolymer in the printed and salt-leached scaffolds, pronounced differences were observed during the course of degradation (Fig. 4b). Initially, from postprocessing to Day 4, the *M*_*n*_ of PLATMC decreased by 43% in the printed scaffold (62 ​kg ​mol^−1^ to 36 ​kg ​mol^−1^) compared to 18% in the salt-leached scaffold (128 ​kg ​mol^−1^ to 92 ​kg ​mol^−1^). From Day 4 until Day 56, the *M*_*n*_ of PLATMC in the printed scaffold decreased by 75% (36 ​kg ​mol^−1^ to 9 ​kg ​mol^−1^) and by an additional 33% until Day 180 (6 ​kg ​mol^−1^). Instead, an inverse trend was observed for PLATMC in the salt-leached scaffold. *M*_*n*_ decreased by 21% from Day 4 until Day 56 (92 ​kg ​mol^−1^ to 73 ​kg ​mol^−1^) and then by an additional 73% until Day 180 (20 ​kg ​mol^−1^). A similar trend was observed for the decrease in *M*_*w*_ over time (Fig. S6). These results demonstrate that the overall degradation rates were similar for PLATMC in the printed and salt-leached scaffolds, despite having different starting *M*_*n*_ values, and suggest that the effects of chain scission on the polymers therefore differed.

#### 2.2.4 Effect of the scaffold fabrication method on the bimodal distribution of PLATMC during the *in vivo* degradation period

The apparent differences in molar mass changes of PLATMC in the printed and salt-leached scaffolds prompted us to make a more detailed evaluation of the *in vivo* degradation. The natures of the GPC traces were therefore compared. Initially, the GPC distribution was unimodal, with a shoulder of small-molar mass species in both scaffold types (Figs. S8 and S9). Over time, the distribution became bimodal, with distinct populations of high- and low-molar-mass species, indicative of predominantly random chain scission compared to chain end scission [6]. The evolution in bimodality is likely a consequence of limited diffusion around the scaffolds by surrounding tissues, restricting the elimination of small oligomeric species. To elucidate this, we approximated the fractional populations from the peak area of the GPC traces and characterized the individual peaks (Fig. 4c). On Days 4 and 56, ≈10–20% of the bimodal curve was represented by small chain fractions in both the printed and the salt-leached scaffolds. On Day 180, ≈40% of the polymer species were represented by the small chain fraction in the printed scaffolds, and ≈30% of the polymer species were represented by the small chain fraction in the salt-leached scaffolds. The average molar mass of the small chain fractional population was consistently between 0.5 and 0.8 ​kg ​mol^−1^, regardless of the time point or scaffold type. These results demonstrate that initially, the accumulation of low-molar mass species did not vary to any great extent between the two scaffold types, although as degradation progressed, larger fractions of low-molar mass species accumulated in the printed scaffold than in the salt-leached scaffold.

#### 2.2.5 Effect of the scaffold fabrication method on the *in vivo* degradation mechanism

The relative rate of diffusion within a material in relation to the rate of hydrolytic cleavage of the polymer dictates the erosion mechanism (Fig. 4a) [59]. When the kinetics of hydrolytic cleavage exceed that of water diffusion, erosion on the surface of the material typically occurs with a constant rate. This creates heterogeneous degradation because hydrolytic cleavage is confined to the outer surface while the interior remains the same (hence resulting in a bimodal GPC trace if diffusion is limited). Conversely, if the kinetics of water diffusion exceed those of hydrolytic cleavage, degradation will occur from the bulk. Degradation then occurs at a uniform rate throughout the polymer matrix. However, the acidic environment created upon chain scission of polyesters complicates these idealized scenarios because of its potential to act autocatalytically. Degradation of a polymer with purely and strong autocatalytic (random) chain scission accelerates as the concentration of acidic chain ends increases, displaying a delayed decrease in *M*_*n*_ over time [56]. Instead, ideal noncatalytic chain scission results in a linear decrease over time. We observed that PLATMC in the salt-leached scaffold had a linear decrease in *M*_*n*_ over time, while PLATMC in the printed scaffold had an initial accelerated reduction in *M*_*n*_ (Fig. 4d). These differences are likely governed by two properties originating from the different fabrication methods: the initial *M*_*n*_ of PLATMC postprocessing and the architectural features of the two scaffold types. The initial greater reduction in *M*_*n*_ for PLATMC in the printed scaffold compared to that for the salt-leached scaffold is likely a result of their differences in initial molar mass in combination with an autocatalytic mechanism. Lower-molar mass polyesters require a smaller number of chain scissions to form water-soluble degradation products, and more acidic chain ends are present for low-molar mass polymers, thereby enabling higher water uptake ability. Consequently, the degradation rate increases. While strong autocatalytic degradation kinetics generally result in an initial delay in the reduction of molar mass (because acceleration occurs when sufficient chain ends have been built up) [56], we observed the opposite trend for PLATMC in the printed scaffold because the polymer had substantially degraded during the scaffold fabrication step prior to implantation (*i*.*e.*, the initial *M*_*n*_ decreased from 146 ​kg ​mol^−1^ to 62 ​kg ​mol^−1^). This likely resulted in an accumulation of acidic chain ends within the scaffold prior to the first timepoint, enhancing the degradation rate through autocatalysis in the bulk already from the start. This would offset the kinetics so that it appeared as a weak autocatalytic mechanism rather than a strong autocatalytic mechanism [56], explaining the initial accelerated reduction in *M*_*n.*_ Even though substantial accumulation of chain ends enhances the water uptake ability, the autocatalytic environment restricts the buffering capacity within the bulk of the scaffold, and as a result of the different degradation environment within the center and at the surfaces of the scaffold, the molar mass broadens toward a bimodal distribution.

**Fig. 5:**
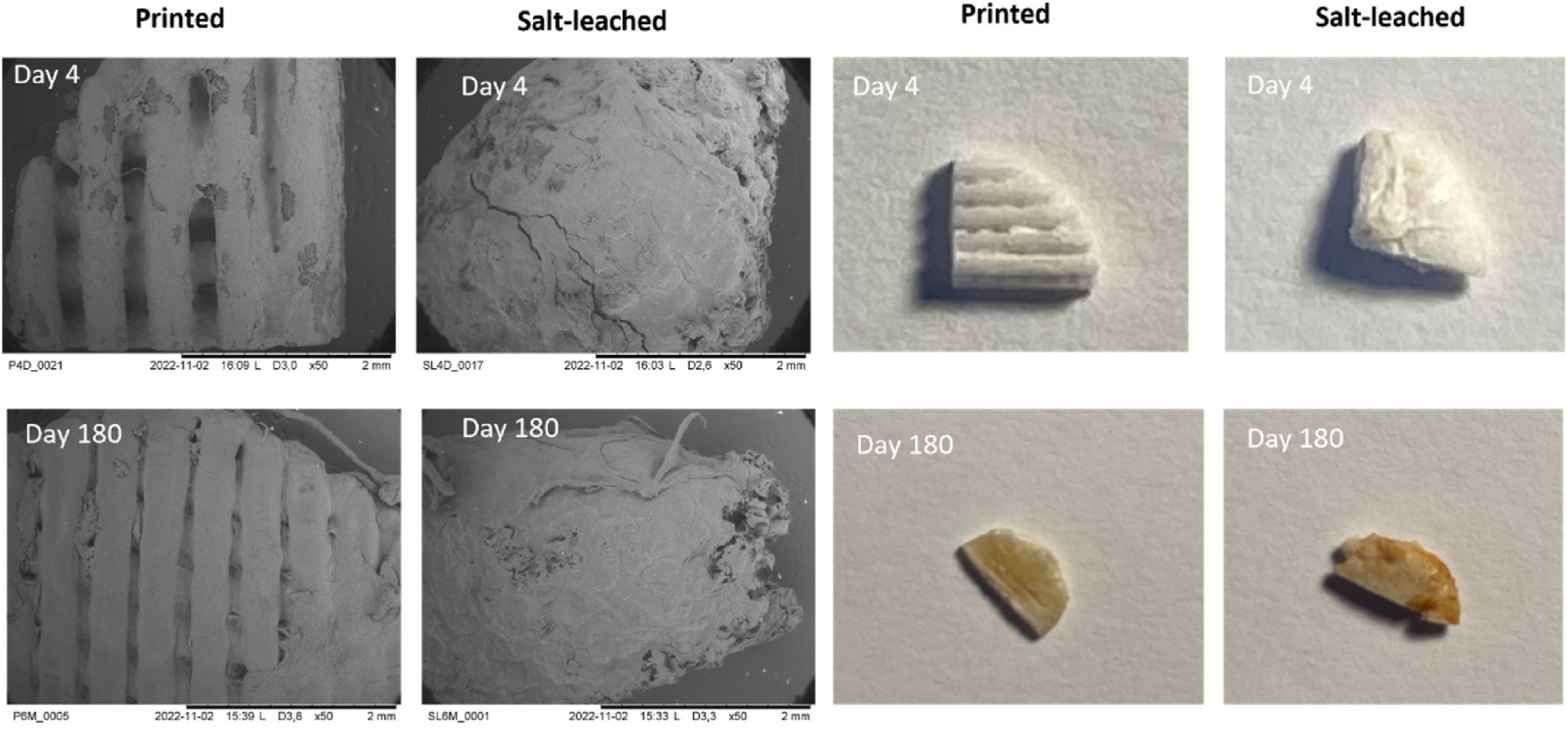
Representative images of printed and salt-leached PLATMC scaffolds during the *in vivo* degradation period. Scaffolds are grouped into their respective processing methods: extrusion-based 3D printing (printed or P) and salt-particulate leaching (salt-leached or S). Values were obtained after explantation of the scaffolds on Days 4 and 180. a) SEM images of printed and salt-leached PLATMC scaffolds on Days 4 and 180 using a magnification of ×50. b) Visual representation of printed and salt-leached PLATMC scaffolds on Days 4 and 180.

Instead, the linear decrease in the *M*_*n*_ of PLATMC in the salt-leached scaffold suggested that degradation occurred through noncatalytic chain scission [56]. This is likely governed by the different architectural features of the salt-leached scaffold compared to the printed scaffold. While the salt-leached scaffold has substantially greater porosity (92% compared to 49% in the printed scaffolds) [19,52] the interconnected porous structure within the salt-leached scaffold has a more complex nature and a smaller gaps as revealed by visual representation of the scaffolds after explantation together with the SEM images (Fig. 5 and Figs. S10–S12). This likely causes slow ingress of water into the salt-leached scaffold, which increases the amount of surface-to-bulk erosion, resulting in an offset of the autocatalytic kinetics and resulting in apparent noncatalytic chain scission [6,59]. Due to the restricted diffusion capacity, the initial degradation rate was lower than that of PLATMC in the printed scaffold (Fig. 4d). Later, when sufficient water uptake and chain cleavage occurred, deformations within the scaffold architecture likely enabled greater diffusion of lower-molar mass species, resulting in an increase in the degradation rate compared to that of the printed scaffold. Although scaffold porosity as a measure is typically used to highlight its importance for the diffusion capacity of polyester-based scaffolds [16,21], (‘porosity’ is, for example, used in mathematical models related to degradation [6,56]), our results demonstrate that porosity alone cannot be related to diffusion capacity but rather the interconnectivity of the pores and the gap sizes within the scaffold architecture.

Taken together, these results suggest that both scaffold types exhibited random chain scission with a restricted diffusion capacity around the scaffolds, as revealed by the bimodal nature of the polymer populations. Autocatalytic degradation kinetics were pronounced for PLATMC in the printed scaffold with an accelerated reduction in *M*_*n*_ due to the substantial presence of acidic chain ends from the scaffold processing method. The linear decrease in *M*_*n*_ for PLATMC in the salt-leached scaffold was likely a consequence of reduced water uptake ability due to the complex pore interconnectivity and small gaps within the scaffolds, resulting in apparent surface erosion kinetics.

#### 2.2.6 Changes in the thermal properties of PLATMC during the *in vivo* degradation period

Having established the changes in molar mass during the *in vivo* degradation period, we determined the thermal properties of the material to expand our understanding of the degradation behavior. While the initial molar mass evidently played a key role in degradation, the crystallinity of a polymer largely dictates its water uptake and diffusion and may therefore be the primary contributor when present [21]. Poly(trimethylene carbonate) (PTMC) is a completely amorphous polymer with a glass transition temperature (*T*_*g*_) typically reported to be approximately −15 to −20 ​°C, while poly(l-lactide) PLLA is semicrystalline, with a *T*_*g*_ commonly in the range of 50–65 ​°C and a *T*_*m*_ of 130–190 ​°C [[61], [62], [63]]. The crystalline structure of PLATMC is therefore determined by the arrangement of the l-lactide (LLA) chain segments, while the *T*_*g*_ is a feature of the amorphous phase largely dictated by the trimethylene carbonate (TMC)-rich regions. The amorphous region of a polymer has greater chain mobility than crystalline regions, thus giving rise to a larger free volume. This in turn translates to the ability for water molecules to penetrate the amorphous phase, causing chain scission and, as a result, being preferentially degraded compared to the crystalline phase [24,25]. The increase in chain mobility then facilitates the crystallization process within the amorphous phase, and consequently, the crystallinity often increases over the degradation time [59]. We therefore determined thermal properties such as *T*_*g*_, melting temperature (*T*_*m*_), crystallinity content (*X*_*c*_) and the peak temperature at which 5% weight loss occurred (*T*_*5%*_) for PLATMC in the printed and salt-leached scaffolds on Days 4, 56 and 180. Prior to scaffold fabrication, PLATMC was semicrystalline, with a *T*_*g*_ of 32 ​°C, a *T*_*m*_ of 158 ​°C and an *X*_*c*_ of 20%. Single glass transitions and melting peaks were obtained throughout the degradation time.

#### 2.2.7 Changes in the thermal degradation of PLATMC during the *in vivo* degradation period

**Fig. 6:**
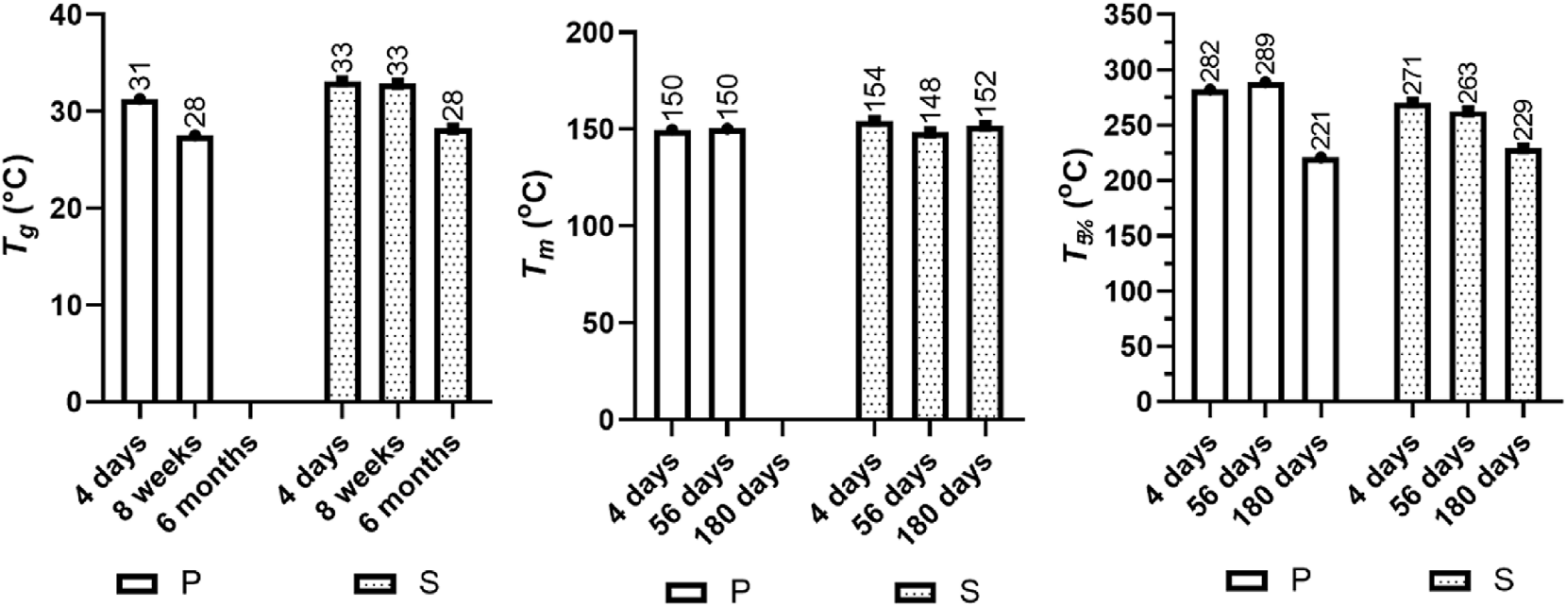
Changes in the thermal properties of PLATMC: glass transition temperature (*T*_*g*_); melting peak temperature (*T*_*m*_); and temperature at which 5% mass loss was observed (*T*_*5%*_). *T*_*g*_ and *T*_*m*_ values were obtained from DSC analysis, and *T*_*5%*_ values were obtained from TGA analysis of the scaffold only. Scaffolds are grouped into their respective processing methods: extrusion-based 3D printing (printed or P) and salt-particulate leaching (salt-leached or S). Values were obtained after explantation of the scaffolds on Days 4, 56, and 180.

A single weight loss step was observed in the thermogravimetric curves for all samples, while earlier onset of thermal degradation was apparent during later stages of the *in vivo* degradation period (Fig. S15a). The charring residues were between ≈0 and 15% and increased over the degradation time. The peak temperatures at which 5% weight loss had occurred (*T*_*5%*_) were similar for the printed and salt-leached scaffolds over time (Fig. 6). Printed scaffolds displayed little variation in *T*_*5%*_ between Days 4 and 56 (282 and 289 ​°C), although on Day 180, *T*_*5%*_ was substantially lower (221 ​°C). A similar trend was observed for the salt-leached scaffolds; only slight differences in *T*_*5%*_ were observed between Days 4 and 56 (271 ​°C–263 ​°C) compared to a larger difference in *T*_*5%*_ on Day 180 (229 ​°C). The difference in weight loss temperature over time is consistent with the reduction in the molar mass of PLATMC over the degradation period. The free volume increases with lower-mass polymers, enabling facile permeability and diffusion of oxygen, water vapor or other volatiles compared to high-molar mass polymers.

#### 2.2.8 Changes in the amorphous and crystalline phases of PLATMC during the *in vivo* degradation period

No major change in *T*_*m*_ was observed in the degradation study for either PLATMC in the printed or salt-leached scaffold (Fig. 6). A slight decrease in *T*_*m*_ was observed at Day 4 for PLATMC in the printed (150 ​°C) and salt-leached scaffolds (154 ​°C), suggesting that the segments in the crystalline phases were more organized in the polymer prior to scaffold fabrication. There was no difference in *T*_*m*_ between Days 4 and 56 for PLATMC in the printed scaffold (150 ​°C), while no data could be obtained at Day 180, likely due to the low molar mass of the polymer resulting in segments that were too short to crystallize. Only a small fluctuation in *T*_*m*_ was observed for PLATMC in the salt-leached scaffold over the course of the degradation study, suggesting little difference in how the segments in the crystalline phase were organized.

Although a crystallization peak was observed in most cases, the degree of crystallinity obtained was less than 1% (based on the theoretical calculation using 100% crystalline PLLA [64]). The low degree of crystallinity reflects the low enthalpy of crystallization (*ΔH*_*c*_) and fusion (*ΔH*_*m*_) observed (Fig. S13). This correlates well to the small variation in *T*_*m*_ over time and suggests limited ability for the chain segments to be organized through crystallization. Restricted movement created upon cooling the scaffolds postprocessing likely prevented crystallization from occurring. A slight increase in *ΔH*_*m*_ was observed between Days 4 and 56 for PLATMC in the printed scaffolds and between Days 56 and 180 for PLATMC in the salt-leached scaffolds. This correlates well to the large decrease in the molar mass of the copolymer from the printed scaffold between Days 4 and 56, while the largest decrease in molar mass was observed between Days 56 and 180 for the copolymer in the salt-leached scaffold. The high degree of hydrolytic cleavage of PLATMC during these respective timepoints results in facile chain shortage, which increases the mobility of the chains and aids in the swelling of the amorphous phase [59]. This allows the chains to reorganize and recrystallize, thereby increasing *ΔH*_*m*_.

The *T*_*g*_ decreased for PLATMC in both the printed and salt-leached scaffolds over the course of the *in vivo* degradation time (Fig. 6). The *T*_*g*_ at Day 4 was slightly lower for PLATMC in the printed scaffold, 30 ​°C compared to 33 ​°C for PLATMC in the salt-leached scaffold. At Day 56, the *T*_*g*_ of PLATMC in the printed scaffold decreased to 25 ​°C, while the *T*_*g*_ of PLATMC in the salt-leached scaffold remained at 32 ​°C. On Day 180, we could not obtain a *T*_*g*_ value for PLATMC in the printed scaffold, likely due to the low molar mass of the copolymer. The *T*_*g*_ of PLATMC in the salt-leached scaffold decreased to 29 ​°C at the same time point. The observed decrease in *T*_*g*_ over the degradation time is likely related to the decrease in molar mass over time for PLATMC in both the printed and the salt-leached scaffolds and explains the faster decrease in *T*_*g*_ for the copolymer in the printed scaffold compared to that in the salt-leached scaffold. Diffusion of water into the scaffolds, followed by chain scission that results in short oligomeric chains and water being present within the scaffold, can act as a plasticizer and consequently lower the value of *T*_*g*_ [24,25]. A further decrease in crystallinity allows for more facile migration of oligomers into bulk, continuously decreasing the value of *T*_*g*_ [59]. These results demonstrate that the thermal properties of the material were not affected to a great extent despite the extensive reduction in *M*_*n*_, illustrating an important aspect for the development of materials where long-term degradability is desirable.

#### 2.2.9 Changes in the chemical composition of PLATMC during the *in vivo* degradation period

**Fig. 7:**
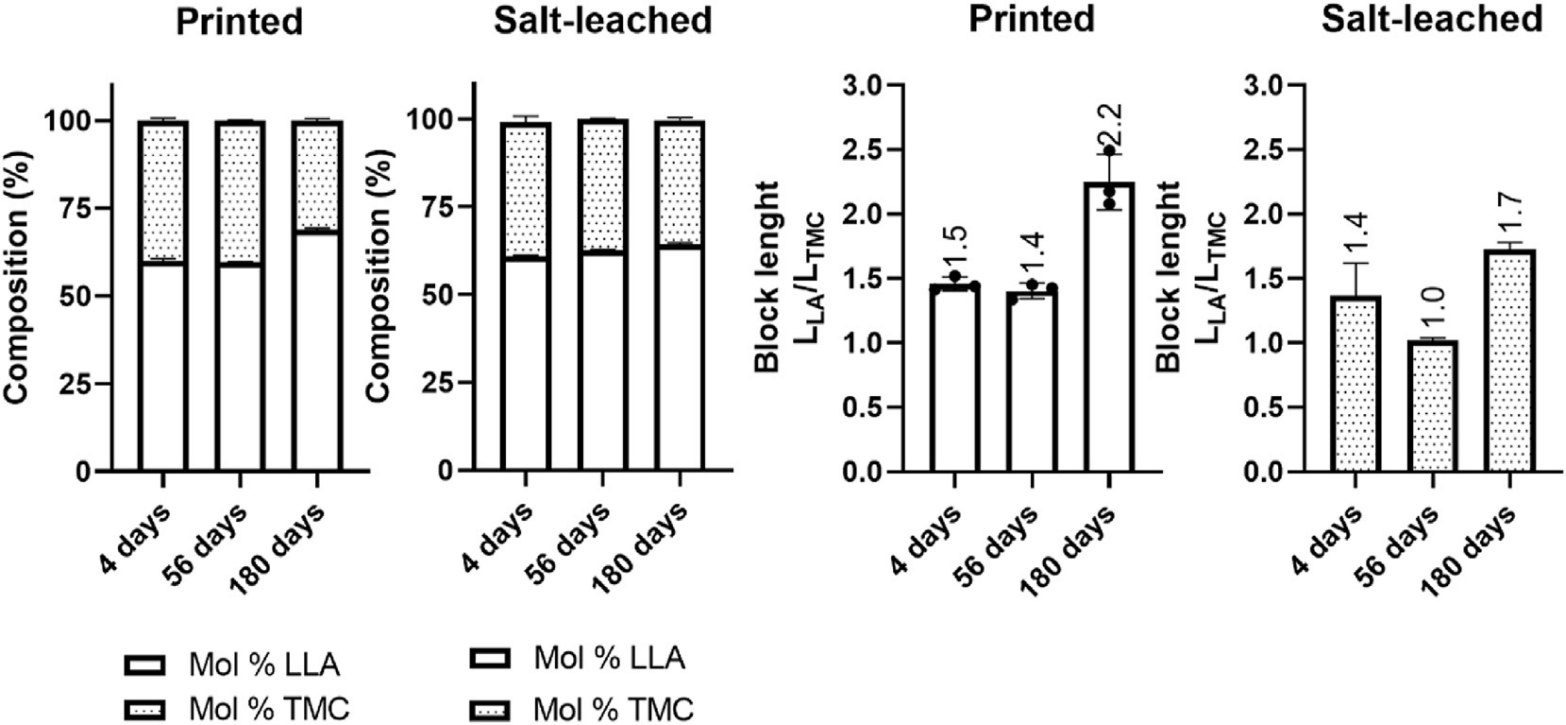
Changes in the polymer microstructure of PLATMC during the *in vivo* degradation period. Scaffolds are grouped into their respective processing methods: extrusion-based 3D printing (printed or P) and salt-particulate leaching (salt-leached or S). Values were obtained after explantation of the scaffolds on Days 4, 56, and 180. a) Changes in the chemical composition of PLATMC. Polymer composition is represented as mol% LLA and TMC. b) Average chain length (L_LA_ and L_TMC_) for PLATMC. Values were obtained from the scaffold using only ^1^H and ^13^C NMR.

The inherent chemical structure of the polymer determines the hydrolytic bond cleavage ability and translates into macroscopic features such as hydrophilicity, solubility, and crystallinity. The chemical composition affects the hydrophilicity of the material and, as a consequence, its water solubility, uptake and diffusion capacity. Furthermore, monomer distribution within a copolymer largely influences its crystallinity [21], which in turn governs the degradation behavior through its ability to take up water. Therefore, we determined the chemical composition and average block length of PLATMC on Days 4, 56, and 180 using Equations (6), (7). ^1^H NMR analysis of PLATMC showed little variation in monomer composition over time (Fig. 7). Prior to scaffold fabrication, the polymer composition of PLATMC was 60 ​mol% LLA and 40 ​mol% TMC, and the average LLA block length (*L*_*LA*_) was 2.2, while the average TMC block length (*L*_*TMC*_) was 1.7 (Fig. S14). No difference in the monomer compositions of PLATMC in the printed and salt-leached scaffolds was observed until Day 56 (Fig. 7). A slight increase in LLA content was observed for PLATMC in the printed scaffold by Day 180, while little difference in monomer composition was observed for the copolymer in the salt-leached scaffold. Consistently, little variation was observed in block length between Days 4 and 56, while the block length ratio *L*_*LA*_*/L*_*TMC*_ increased for PLATMC both in the printed and in the salt-leached scaffolds between Days 56 and 180. *L*_*LA*_ increased from 2.5 to 3.0 over the degradation time for PLATMC in the printed scaffold, while *L*_*TMC*_ decreased from 1.7 to 1.3 (Fig. S14). The change was smaller for PLATMC in the salt-leached scaffold, where *L*_*LA*_ increased from 2.4 to 2.6 over time, while *L*_*TMC*_ decreased from 1.8 to 1.5. The short *L*_*LA*_ block length observed throughout the degradation period likely prevented facile crystallization from occurring and thereby explained the low crystallinity obtained.

These results are consistent with the results obtained from the thermal analysis, suggesting little difference in thermal properties throughout the degradation period, except for PLATMC in the printed scaffold on Day 180. The crystallinity in PLATMC is governed by rich LLA segments, while TMC segments exhibit a more flexible nature [[61], [62], [63]]. The rich TMC segments in PLATMC are expected to be more prone to water uptake and thereby chain cleavage because of their amorphous nature. The increase in LLA content accompanied by an increased *L*_*LA*_ for PLATMC in the printed scaffold suggests that the carbonate linkages within the TMC segments were more susceptible to hydrolytic cleavage compared to the ester linkages within the LLA segments, as expected. This was supported by the complete disappearance of the TTT triad sequence in the carbonyl region of the ^13^C NMR spectrum (155.1 ​ppm) after 180 days, while the TLL triad sequence remained (154.4_TMC_ ppm). PLATMC in the salt-leached scaffold displayed consistent presence of the TTT triad sequence in the carbonyl region, in accordance with the smaller variation in *L*_*LA*_ and *L*_*TMC*_ block length over time. This is consistent with higher water uptake for the printed scaffold compared to the salt-leached scaffold as a result of their different degradation environments and scaffold architectures lowering *M*_*n*_ and *T*_*g*_.

#### 2.2.10 Time frame of the *in vivo* degradation of the hybrid hydrogel scaffold

Tissue remodeling is a dynamic process operating over diverse time spans depending on the tissue type and its interaction with the material. Materials that do not meet the gradual tissue formation process may lead to insufficient remodeling if degradation of the material occurs too fast or tissue damage if the material degrades slowly. Therefore, it is important for scaffold materials to successively degrade throughout the process of tissue formation, and understanding the timeframe of degradation characteristics of the material is vital for successful clinical outcomes. Resorption of polyesters occurs by the formation of oligomers or low-molar mass species, which are able to diffuse through the material and through further dissolution. Our results demonstrate that regardless of the scaffold fabrication method, within 180 days, the degradation products exhibited masses between 5 and 35 ​kg ​mol^−1^, which is desirable since the formation of degradation products of masses below 30 ​kg ​mol^−1^ is crucial for renal clearance [21]. Visual inspections as well as SEM images of the explanted scaffolds revealed that the scaffolds remained relatively stable over the degradation period with no larger cracks or holes appearing throughout the time (Fig. 5 and Figs. S10–S12). The surface was smoothened throughout the degradation period for both scaffold types. The salt-leached scaffold appeared smoothened already on Day 4 while the printed scaffolds maintained defined strands at least until Day 56. Both scaffold types exhibited slightly more granulated nature on Day 180. Since the scaffolds were in a rubbery state throughout the degradation period, as revealed by the observed *T*_*g*_, the rate of degradation was likely enhanced. A similar time frame of degradation has been observed for other PLATMC-based biomaterials. Complete degradation was observed after 180 days for the *in vivo* degradation of PLATMC films implanted subcutaneously into rats [27]. Similarly, the *in vivo* degradation of PLATMC-based occluders (*M*_*n*_ of 56 ​kg ​mol^−1^; composition of 70 ​mol % LLA and 30 ​mol % TMC, and *T*_*g*_ of 41 ​°C) subcutaneously implanted in the backs of rabbits completely degraded within 120 days [28]. The fact that the crystallinity of PLATMC did not increase over time within the scaffolds and that the *T*_*g*_ remained below body temperature throughout the degradation study suggests that the scaffolds remained flexible and adaptable throughout the degradation period and that the polymer being in a rubbery state during the degradation period resulted in a favorable degradation time.

## 3 Conclusions

The results presented herein demonstrate the ability to develop a concurrent hybrid material exhibiting short-term tissue-relevant properties without impeding long-term structural integrity and mechanical properties. The hybrid material rendered a desirable cell-instructive “local” elastic modulus governed by the transparent and minimally swollen bio-orthogonally crosslinked hyaluronan hydrogel. Long-term degradability over 180 days *in vivo* was realized using PLATMC scaffolds processed through salt-particulate leaching or extrusion-based 3D printing. Importantly, the unaffected degradation behavior of PLATMC by the inclusion of the hydrogel was observed even though the polymer in the printed scaffold underwent a typical autocatalytic bulk degradation mechanism, illustrating the favorable physicochemical properties of the hydrogel. Notably, PLATMC in the salt-leached scaffold exhibited uniform degradation kinetics illustrative of noncatalytic chain cleavage with surface eroding-like properties, a behavior typically not seen in polyester-based scaffolds. The effect of the processing method on the material properties markedly extends its degradability, emphasized by the distinctly different degradation mechanisms of PLATMC in the printed and salt-leached scaffolds. The initial molar mass and prevalence of chain cleavage of the polymer postprocessing together with architectural features such as pore interconnectivity and gap size within the scaffold constructs are fundamental to the degradation characteristics. This study extends and signifies the current understanding of how the processing method affects the *in vivo* degradability of polyester-based materials, a vital aspect for successful clinical outcomes. The results presented convey that the hybrid HA hydrogel PLATMC scaffold is a promising material for the development of cell-instructive microenvironments for tissue engineering applications with the potential to act as a drug and cell delivery platform where long-term degradability is desirable.

## 4 Experimental

### 4.1 Materials used

Hyaluronan sodium salt from Streptococcus equi (bacterial glycosaminoglycan polysaccharide; *M*_*n*_ of 1500–1800 ​kg ​mol^−1^), 4-(4,6-dimethoxy-1,3,5-triazin-2-yl)-4-methylmorpholinium chloride (DMTMM; ≥96.0%), 1,8-bismaleimido-diethyleneglycol (BM(PEG)_2_), dialysis bags (35 ​mm, MWCO of 12 ​kg ​mol^−1^), furfurylamine (≥99%), and hyaluronidase (from bovine testes, Type I–S, 400–1000 units mg^−1^ solid) were purchased from Merck/Sigma‒Aldrich®. Sodium chloride (Extra Pure SLR) and morpholineethanesulfonic acid monohydrate (MES, 98%) were obtained from Fisher Scientific. Medical grade poly(*l*-lactide*-co-*trimethylenecarbonate) (Resomer® LT706S; 60 ​mol% LLA/40 ​mol% TMC; *M*_*n*_: 146 ​kg ​mol^−1^, Ð: 1.5; IV: 1.2–1.6, Evonik Industries) was stored at −20 ​°C under a nitrogen atmosphere prior to use. Hyaluronan and its derivatives were stored at −20 ​°C under a nitrogen atmosphere prior to use. DMEM culture medium and penicillin/streptomycin were obtained from Invitrogen (Carlsbad, CA, USA).

### 4.2 Synthetic procedures and characterizations of the hybrid hydrogel scaffold

#### 4.2.1 Furan modification of hyaluronan

The procedure was adapted from previous literature [35]. To a 100 ​mL round bottom flask containing MES buffer (40 ​mL, 100 ​mM, pH 5.5), was added hyaluronan sodium salt (0.4 ​g, 1 equiv. COOH) followed by the addition of DMTMM (0.56 ​g, 2 equiv.). The reaction was left stirring for 10 ​min before furfurylamine (95 ​μL, 1 equiv.) was added dropwise. The reaction was left at room temperature for 24 ​h and dialyzed against MQ H_2_O over 3 days (MWCO 12 ​kg ​mol^−1^). The mixture was allowed to freeze at −20 ​°C for 12 ​h and then freeze-dried over 3 days. The degree of substitution was confirmed by ^1^H NMR by comparing the integration from the furan-proton at 7.53 ​ppm and that from the N-acetyl protons on hyaluronan at 2.04 ​ppm (Fig. S1).

#### 4.2.2 Fabrication of hybrid hydrogel scaffolds through Diels-Alder crosslinking

Furan-modified hyaluronan was dissolved in 100 ​mM MES (pH 5.5; final concentration of 0.5 w/v%) over 4 ​h before it was added to a 96-well plate containing PLATMC scaffolds (400 μL/scaffold). The well-plates were placed on a shaker to solubilize after which the crosslinking agent BM(PEG)_2_ (1 equiv. maleimide to furan-groups; 100 μL/scaffold in 100 ​mM MES buffer; sonicated for 45 ​min prior to use) was injected into the scaffolds and then incubated at 37 ​°C for 24 ​h. For all conjugation efficiency experiments, the experiments were performed in triplicate using salt-leached scaffolds. The conjugation efficiency was determined based on ^1^H NMR following degradation of the glycosidic bonds of hyaluronan using hyaluronidase (200 U 100 ​μL^−1^ over 2 days at 37 ​°C followed by lyophilization. The conjugation efficiency was evaluated based on two factors: the % of reacted furans per HA by Equation (1) (where DS_initial_ ​= ​60%) and the sum of hydrolyzed or unreacted maleimide groups normalized to reacted furans by Equation (2).

(1) $$Reacted\;furans\;per\;HA\;\left(\%\right):\;\left({DS}_{initial}-{DS}_{gel}\right)\;x\;100\;\%$$

(2) $$Hydrolyzed+unreacted\;maleimides\;per\;HA=\frac{\left(I_{5.97ppm}+I_{6.89ppm}\right)}{I_{2.04ppm}}$$

#### 4.2.3 Swelling and stability study of the hydrogel and hybrid hydrogel scaffolds

To examine the swelling and stability capacity of the hydrogel/scaffolds, samples were synthesized in preweighed vials according to the procedure described. The experiments were performed in triplicate (n ​= ​3) using salt-leached scaffolds. Samples were preweighed to give *M*_*0*_ (initial gel state) and incubated in 1 ​mL of PBS at 37 ​°C. The swelling ratio from the initial gel state to the wet state following further swelling was measured at suitable time intervals and determined by measuring the mass (*M*_*t*_) after removal of the buffer and turning the vials upside down followed by gentle drying of excess solution. The gels were then replenished with fresh buffer. The same experiment was repeated after freeze-drying of the hydrogel/scaffolds (where *M*_*0*_ represents the initial dry state). The weight change was calculated based on Equation (3), and the equilibrium swelling ratio was calculated based on Equation (4).

(3) $$Weight\;change\;\left(\%\right):\;\left(M_{t}-M_{0}\right)\;x\;100\;\%$$

(4) Equilibirumswellingratio(q):(MtM0)

#### 4.2.4 Rheology

The samples used for rheological measurements were prepared in triplicate (n ​= ​3) as described, with a hyaluronan concentration of 5 ​mg ​mL^−1^ (200 ​μL per sample). Briefly, HA_f_ was incubated with BM(PEG)_2_ O.N. at R.T. or 37 ​°C (Fig. 2c), and for comparisons, neat furan-modified HA_f_ or unmodified HA was dissolved in deionized H_2_O (Fig. S5). Rheological measurements were performed using a TA Instruments Discovery HR-2 rheometer with a parallel plate geometry of 25 ​mm in diameter and a Peltier plate. The gap size was 200 ​μm, and the measurements were run at 37 ​°C. A solvent trap was used to prevent the formulations from drying out. Due to the difference in the viscoelastic behaviors of the different gels, the applied pressure was varied while all other parameters were kept constant during measurements (20 ​Pa for HA and HA_f_ samples; 10 ​Pa for HA_f_/PEG formulated at R.T.; 5 ​Pa for HA_f_/PEG at 37 ​°C). An amplitude sweep was initially carried out to determine the linear viscoelastic region at 1 ​Hz from 1 to 100 ​Pa. Frequency sweeps were conducted using small amplitude oscillatory shear over 0.1–20 ​Hz with a 1.0 ​s sampling time. Trios v.4.21 software was used for data acquisition. The apparent average mesh size (*ξ*_*a*_) was estimated using Equation (5) [53], derived from rubber elasticity theory.

(5) $$\xi ={\left(\frac{G^{\prime }N_{A}}{RT}\right)}^{-1/3}$$

where *N*_*A*_ refers to Avogadro's constant, *R* is the gas constant, *T* is the temperature, and *G′* is the storage modulus of the hydrogel.

### 4.3 Polymer degradation characterizations

#### 4.3.1 ^1^H and ^13^C Nuclear Magnetic Resonance (NMR)

NMR was performed on a Bruker Avance Ultrashield™ spectrometer (^1^H: 400.13 ​MHz; ^13^C: 100.62 ​MHz), with the chemical shifts (ppm, *δ*) referenced to the residual solvent peak (CDCl_3_ for PLATMC and D_2_O for HA derivatives). MestReNova software was used for data acquisition. The monomer composition of PLATMC was calculated from ^1^H NMR spectra by comparing the methine proton of the lactidyl unit at 5.16 ​ppm to the methylene proton of the carbonate unit at 4.23 ​ppm. The average block length of the lactidyl units (*L*_*LL*_) and the carbonate units (*L*_*TMC*_) in the copolymer were calculated from the integrated area in the carbonyl region of the ^13^C NMR spectra based on Equations (6), (7) [65].

(6) $$L_{LL}=\frac{1}{2}\;x\;\frac{{\;\left(LLT\right)}_{170.4ppm}+{\;\left(TLL\right)}_{170ppm}+{\left(LLL\right)}_{169.7ppm}}{{\;\frac{1}{2}\times ((LLT)}_{170.4ppm}+{\left(TLL\right)}_{170ppm})}$$

(7) $$L_{TMC}=\frac{{\;\left(TTT+TTL\right)}_{155ppm}+{\;\left(LTT+LTL\right)}_{154.4ppm}\;}{{\;{\left(LTL\right)}_{154.4ppm}+\frac{1}{2}\times ((TTL)}_{155ppm}+{\left(LTT\right)}_{154.4ppm})}$$

#### 4.3.2 Gel Permeation Chromatography (GPC)

The number average and weight average molar mass (*M*_*n*_ and *M*_*w*_) and dispersities (*Ð*) of PLATMC were determined from a GPCMAX system equipped with an RI detector and referenced to polystyrene standards (160–371 ​000 ​g ​mol^−1^). Chloroform was used as the mobile phase (1 ​mL ​min^−1^, 35 ​°C), and flow rate fluctuations were corrected by using toluene as an internal standard. *M*_*n*_, *M*_*w*_ and *Ð* were determined for hyaluronan from a Dionex Ultimate-3000 HPLC system referenced to pullulan standards (342–708 ​000 ​g ​mol^−1^). Sodium hydroxide (100 ​mM) was used as the mobile phase (1 ​mL ​min^−1^, 40 ​°C).

#### 4.3.3 Differential Scanning Calorimetry (DSC)

DSC was used to determine the glass transition temperature (*T*_*g*_), crystallization point temperature (*T*_*c*_), melting peak temperature (*T*_*m*_), enthalpy of fusion (*ΔH*_*m*_) and cold crystallization (*ΔH*_*c*_) of PLATMC. Samples (between 5 and 15 ​mg) were run under nitrogen flow with a heating and cooling rate of 10 ​°C min^−1^. The samples were cooled well below the expected *T*_*g*_ and then reheated, i.e., from −20 ​°C to 220 ​°C, using a Mettler Toledo DSC 1 instrument calibrated with indium. Data are reported from the first heating run. *T*_*g*_ is taken from the midpoint ISO, and the degree of crystallinity (*X*_*c*_) was calculated assuming *ΔH*_*m*_° ​= ​93.0 ​J ​g^−1^ for 100% crystalline PLLA [64].

#### 4.3.4 Thermal Gravimetric Analysis (TGA)

TGA was used to determine the thermal stability of the PLATMC scaffolds. Samples (between 2.1 and 18.1 ​mg) were run under nitrogen flow with a heating rate of 10 ​°C min^−1^ from 25 ​°C to 500 ​°C using a Mettler Toledo TGA/DSC 1 instrument. The flow rate was set to 80 ​mL ​min^−1^. Data are reported as the temperature at which 5% mass loss (*T*_*5%*_) occurred.

#### 4.3.5 Scanning electron microscopy (SEM)

The top surface of the scaffolds was visualized using a TM-1000 tabletop scanning electron microscope (SEM, Hitachi, Japan) with an acceleration voltage of 15 ​kV. No conductive coating was used for the tabletop SEM evaluation. Images were acquired at magnifications of×50, ×100, ×150 and x200.

#### 4.3.6 Scaffold fabrication methods

The scaffolds were fabricated through salt-particulate leaching and extrusion-based 3D printing, as previously described [19]. Briefly, salt-leached scaffolds were fabricated by allowing the solvent to evaporate from PLATMC dissolved in chloroform and blended with sodium chloride salt particles (particle size of 75–500 μ). Circular scaffolds (D: 8 ​mm; H: 1.5–2 ​mm) were punched out, and the salt particles were washed in deionized water. 3D-printed scaffolds were fabricated on a 3D Bioplotter® instrument, EnvisionTEC Germany, by preheating the cartridge to 220 ​°C. PLATMC was added to the cartridge and kept for 4 ​min before the printing temperature was set to 190 ​°C. The inner diameter of the needle was 0.4 ​mm, the outer diameter was 0.7 ​mm, the printing speed was 8–10 ​mm ​s^−1^, and the pressure ranged between 4 and 6 ​bar. A 4-layer sheet was printed, and circular-shaped scaffolds were punched out (D: 8 ​mm; H: 1 ​mm). Scaffolds were stored at −80 ​°C until further use. Scaffolds were then either used after fabrication, immersion-coated using HA as previously described or fabricated into hybrid hydrogel scaffolds as described in ‘Fabrication of hybrid hydrogel scaffolds through Diels-Alder crosslinking reaction’. The same concentration The scaffolds were subjected to −20 ​°C and freeze-dried before they were sterilized by ethylene oxide, which was previously shown to not affect the molar mass of PLATMC [52]. Fig. S16

#### 4.3.7 In vivo degradation

The *in vivo* part of the study was approved by the Norwegian Animal Research Authority (Mattilsynet, FOTS - 17 ​734). Sixty-three healthy, male Lewis rats (6 weeks old, weight: 180 ​g) were used for the *in vivo* degradation assessment as previously described (3–6 rats for each time point) [66]. Four rats were kept in each cage and acclimatized for one week at the Animal Facility, University of Bergen. Four scaffolds were implanted subcutaneously into each animal. Gas anesthesia was supplemented through gas masks, and the head was fixed in a custom-made appliance. A small incision was made along the vertebral column. Using blunt dissection, a pocket was created on both sides of the incision, and one scaffold/cell construct was inserted into each pocket. The incisions were closed with resorbable sutures. Animals were inspected daily. Scaffolds were explanted on Days 4, 56 and 180, and excess tissue surrounding the scaffolds was gently dissected before they were freeze-dried, stored at −80 ​°C and then used for degradation analyses. The scaffolds were cut for sample allocation by utilizing one quarter of each scaffold in the current study for degradation analyses (one quarter of scaffold was used per measurement), while the remaining scaffolds were stored at −80 ​°C for later biological evaluation. The mesenchymal stem cells seeded on the scaffolds were derived from 5 independent donor rats and isolated, cultured and characterized as described previously [67].

### 4.4 Statistical analysis

All statistical analyses were performed using GraphPad Prism version 8.0.2 and are expressed as the mean ​± ​SD. Two-tailed Student's unpaired *t*-test was used to assess differences within two groups (95% confidence level). Ordinary one-way ANOVA Tukey's post-hoc test was used for multiple comparisons of the mean in each group. Statistical significance was defined as follows: N.S. ​= ​not significant, ∗p ​≤ ​0.05, ∗∗p ​≤ ​0.01, ∗∗∗p ​≤ ​0.001, ∗∗∗∗p ​≤ ​0.0001.

## Credit author statement

**Kivijärvi, T**.: Conceptualization, Methodology, Validation, Formal analysis, Investigation, Resources, Data Curation, Writing - Original Draft, Writing - Review & Editing, Visualization. **Goksøyr, Ø.:** Conceptualization, Methodology, Investigation, Resources, Writing - Review & Editing. **Yassin, M. A**.: Conceptualization, Methodology, Resources, Supervision. **Jain, S**.: Resources, Writing - Review & Editing. **Yamada, S**.: Methodology. **Morales-López, A**.: Resources. **Mustafa, K.:** Conceptualization, Resources, Writing - Review & Editing, Supervision, Project administration, Funding acquisition. **Finne-Wistrand, A:** Conceptualization, Resources, Writing - Review & Editing, Supervision, Project administration, Funding acquisition.

## Funding

This work was financially supported by the 10.13039/501100001729Swedish Foundation for Strategic Research (RMA15-0010), the 10.13039/501100005416Research Council of Norway (273551), and the 10.13039/501100004257Helse Vest Funding, Norway (912048).

## Declaration of competing interest

The authors declare that they have no known competing financial interests or personal relationships that could have appeared to influence the work reported in this paper.

## Data Availability

Data will be made available on request.
