# Supplementary material for: Hybrid material based on hyaluronan hydrogels and poly(l-lactide-co-1,3-trimethylene carbonate) scaffolds toward a cell-instructive microenvironment with long-term in vivo degradability
Source: Mater Today Bio. 2022 Nov 1;17:100483. doi: 10.1016/j.mtbio.2022.100483 (PMC9672426; doi:10.1016/j.mtbio.2022.100483)
Supplement: Multimedia component 1 [file mmc1.docx]

SUPPORTING INFORMATION

Hybrid material based on hyaluronan hydrogels and poly(L-lactide*-co-*1,3-trimethylene carbonate) scaffolds towards a cell-instructive microenvironment with long-term *in vivo* degradability

Tove Kivijärvi^1^, Øyvind Goksøyr^2,3^, Mohammed A. Yassin^2^, Shubham Jain^1^, Shuntaro Yamada^2^, Alvaro Morales-López^1^, Kamal Mustafa^2^, Anna Finne-Wistrand^1^

^1^Department of Fibre and Polymer Technology, KTH Royal Institute of Technology, Stockholm, Sweden

^2^Centre of Translational Oral Research (TOR), Department of Clinical Dentistry, University of Bergen, Bergen, Norway

^3^Department of Oral and Maxillofacial Surgery, Haukeland University Hospital, Bergen, Norway


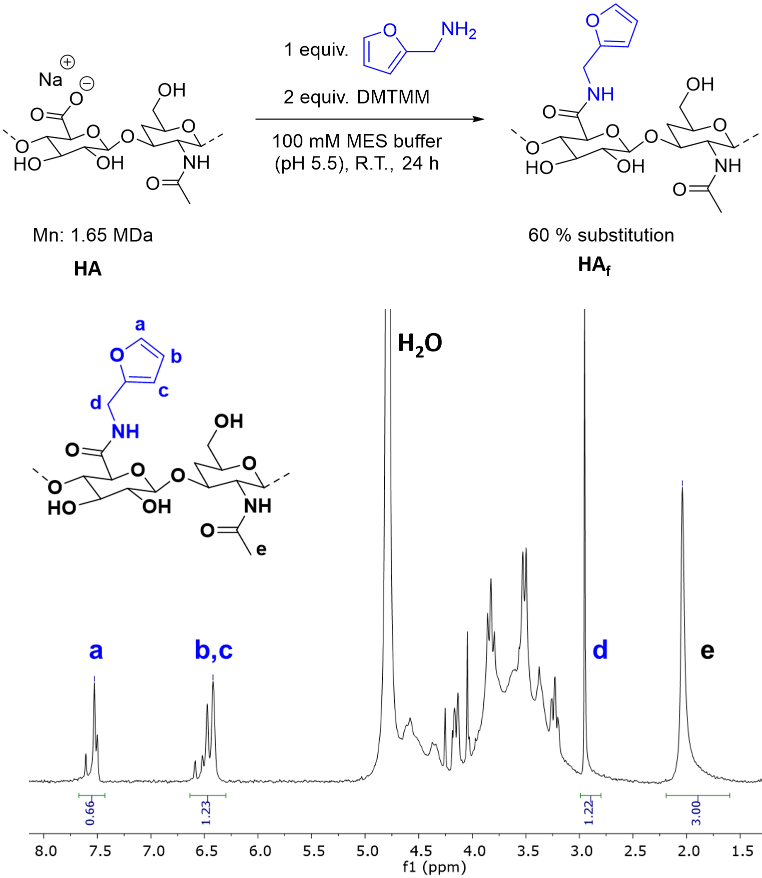
**Figure S1.** Synthesis of furfuryl-modified hyaluronan (HAf). Degree of substitution was determined by 1H NMR comparing the furan proton (a) to the N-acetyl groups (e) on HA.


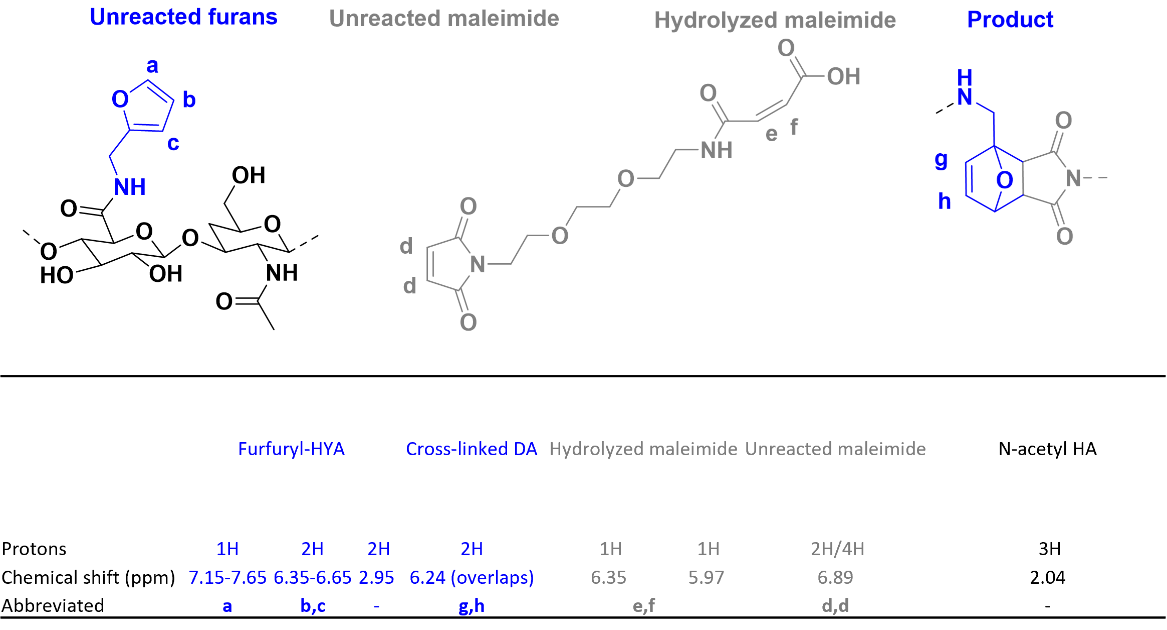
**Figure S2.** ^1^H NMR chemical shifts of unreacted furans, unreacted maleimide and hydrolyzed maleimide used to determine the conjugation efficiency after digestion using hyaluronidase. The chemical shifts of the cross-linked Diels-Alder adduct (product) overlapped and could therefore not be used to quantify the conjugation efficiency. Conjugation efficiency was determined after degrading the samples using hyaluronidase. As control groups, Ha_f_ and BM(PEG)_2_ were also subjected to hyaluronidase separately.


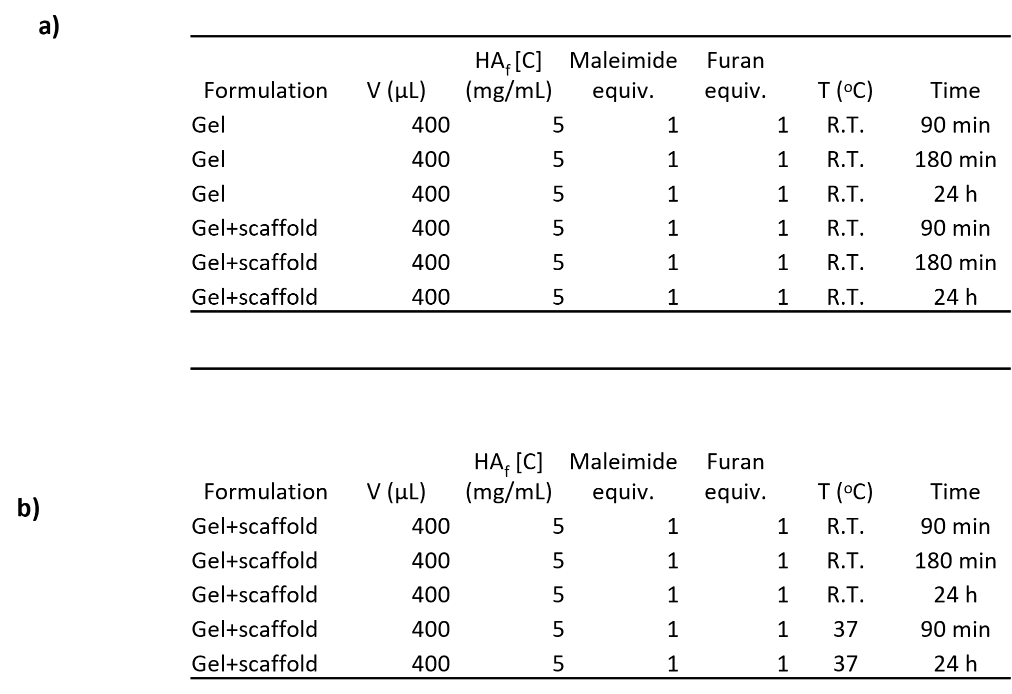

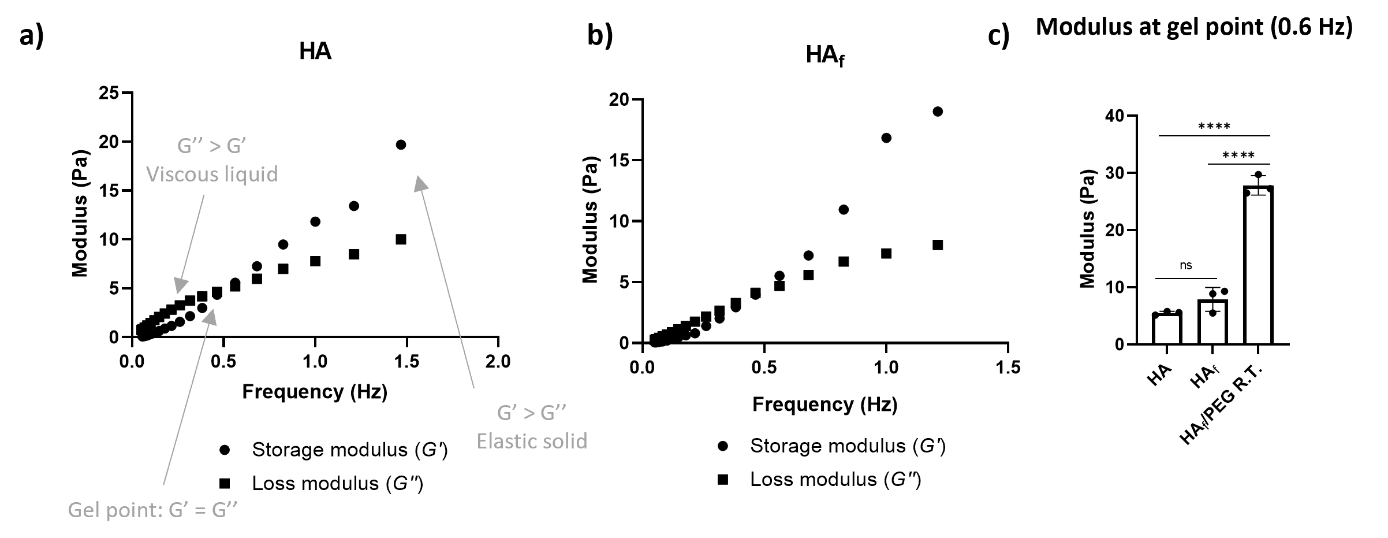
**Figure S3.** Experimental conditions used to determine the conjugation efficiency of the Diels-Alder cross-linking of HA_f_ and BM(PEG)_2_ around PLATMC scaffolds. The tables represents a) time-dependency; b) temperature dependency.

**Figure S4.** Viscoelastic behavior of 5 % w/v hyaluronic acid (HA), furan modified hyaluronic acid (HA_f_), and HA_f_/PEG formulation incubated at R.T. for 24 h. a) Representative frequency sweep of HA and HA_f_ formulations; b) Modulus of HA, HA_f_ and HA_f_ /PEG formulations at gel point during frequency sweep (at 0.6 Hz in all cases). No significant difference was observed between HA and HA_f_, while the modulus was significantly higher for HA_f_ /PEG formulation. Statistical significance was determined using Ordinary one-way ANOVA Turkey’s post-hoc test: N.S. = not significant, *p ≤ 0.05, **p ≤ 0.01, ***p ≤ 0.001, ****p ≤ 0.0001 (n= 3; mean ± SD).


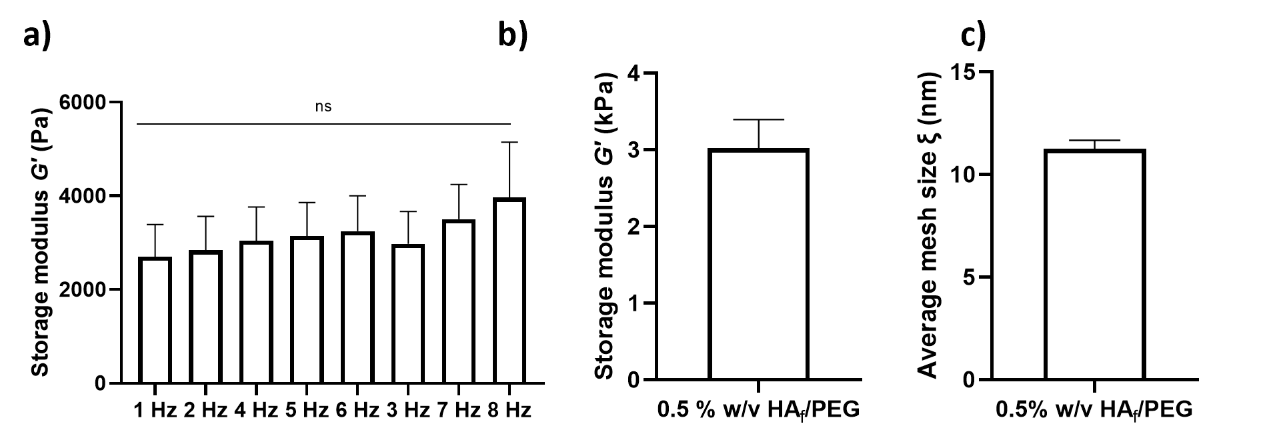
**Figure S5.** a) No significant difference in storage modulus (*G’*) of pre-formed 5.0 % w/v HA_f_/PEG gel over 1-8 Hz frequency sweep; b) Final storage modulus (*G’*) of 5.0 % w/v HA_f_/PEG gel. c) Average mesh size (ξ) estimated from G’. Statistical significance was determined using Ordinary one-way ANOVA Turkey’s post-hoc test: N.S. = not significant, *p ≤ 0.05, **p ≤ 0.01, ***p ≤ 0.001, ****p ≤ 0.0001 (n= 3; mean ± SD).


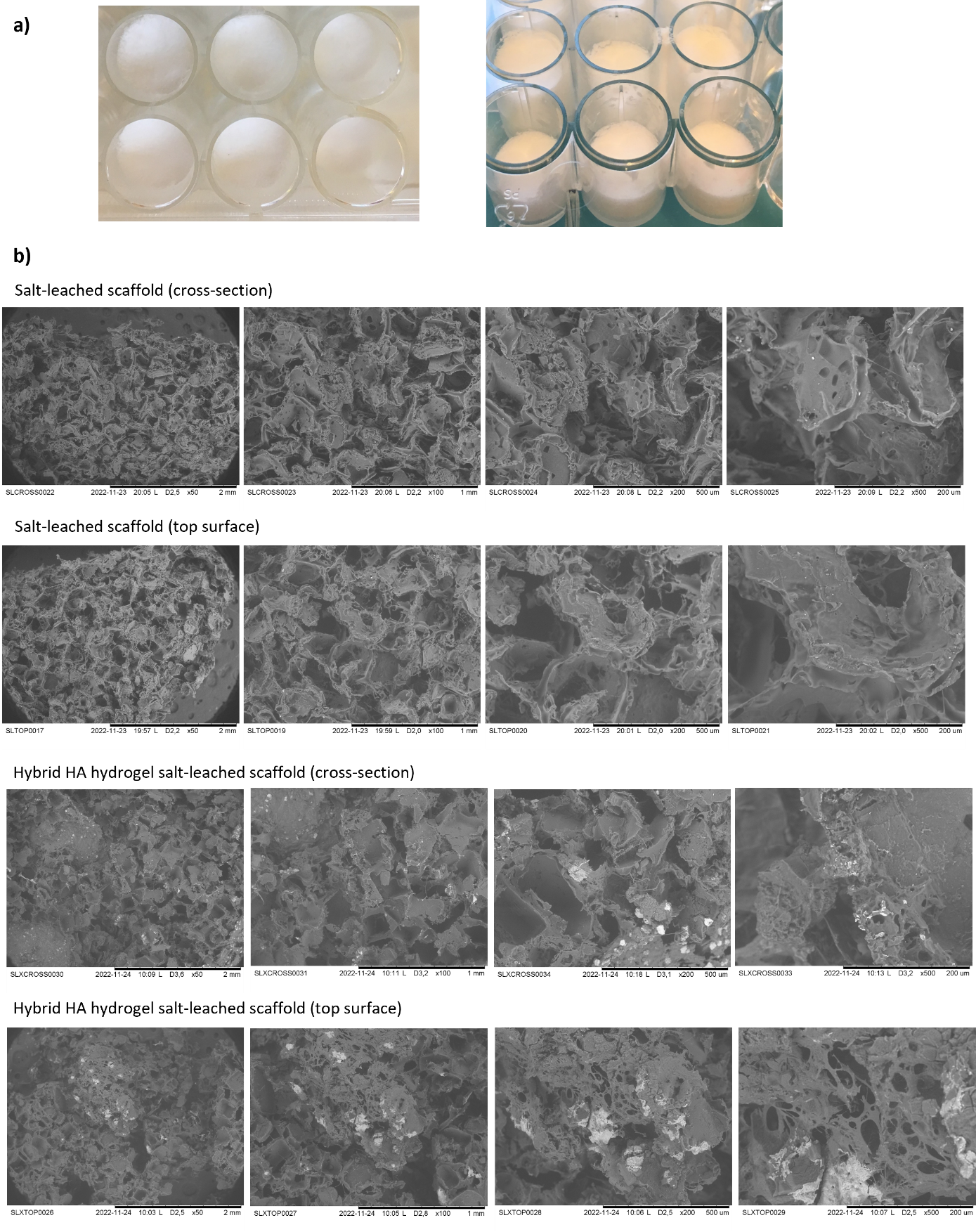


**Figure S6.** a) Visual representation of the formulated hybrid HA hydrogel scaffold (salt-leached scaffolds) after freeze-drying. b) SEM images of salt-leached PLATMC scaffolds with and without HA hybrid hydrogel. The magnification is x50,x100,x200 and x500.


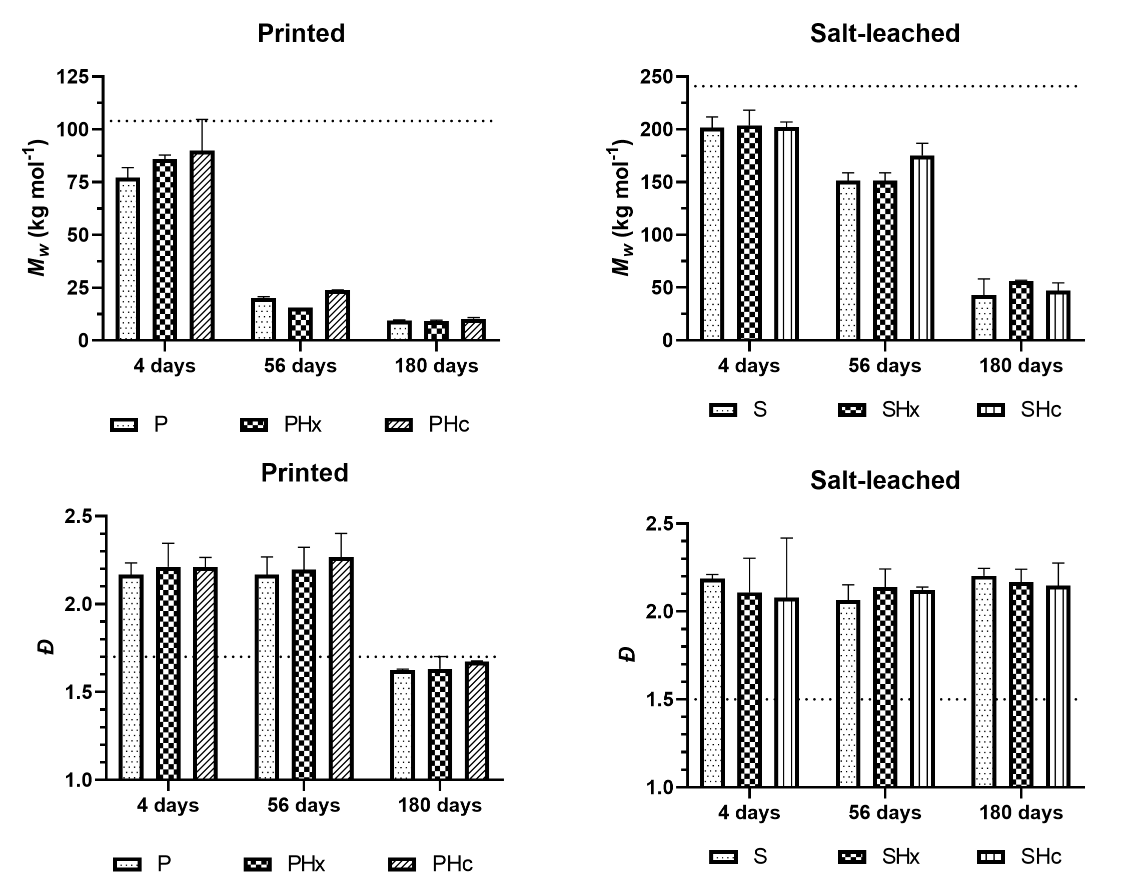
**Figure S7**: Changes in molar mass distribution of PLATMC during the in vivo degradation period. Scaffolds are grouped into their respective processing method; extrusion based 3D-printing (Printed) and salt-particulate leaching (Salt-leached). The evolution ín average molar mass distribution a) Mw, and b) dispersities, analyzed by GPC on day 4, 56, and 180 after explantation of the scaffolds. Values refer the the major peak when the GPC trace displayed a bimodal curve and therefore the dispersity may be misleading. Abbreviations refer to Printed (P); Salt-leached (S); Cells (C); HA (H); cross-linked (X); coated (c). Dashed line represents initial Mw, and D after fabrication of the scaffolds.


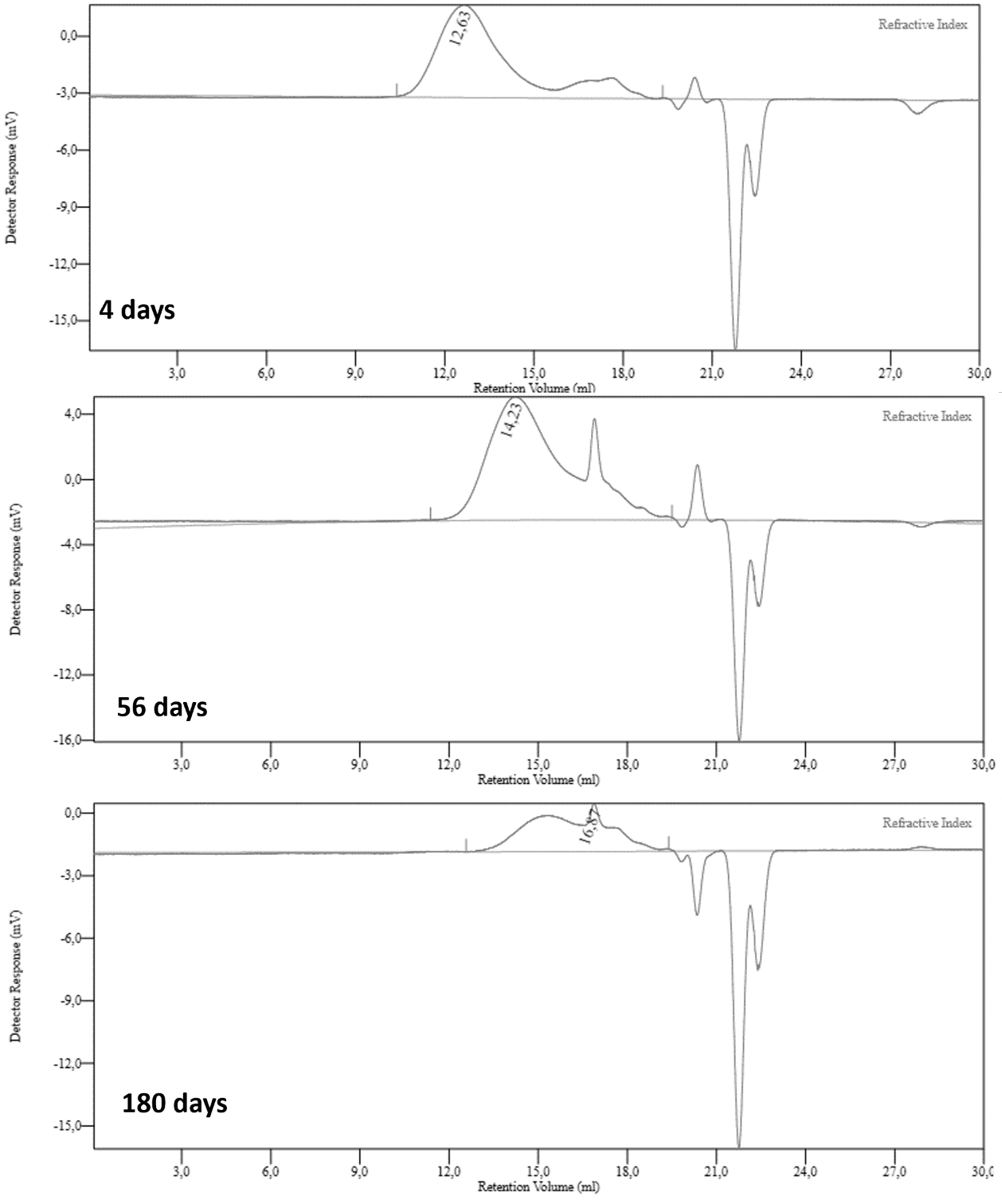
**Figure S8.** Representative evolution of GPC traces over the time of the in vivo degradation period for PLATMC in extrusion based 3D-printed scaffolds. The curves corresponds to time points 4, 56, and 180 days after explantation of the scaffolds and dissolution in chloroform.


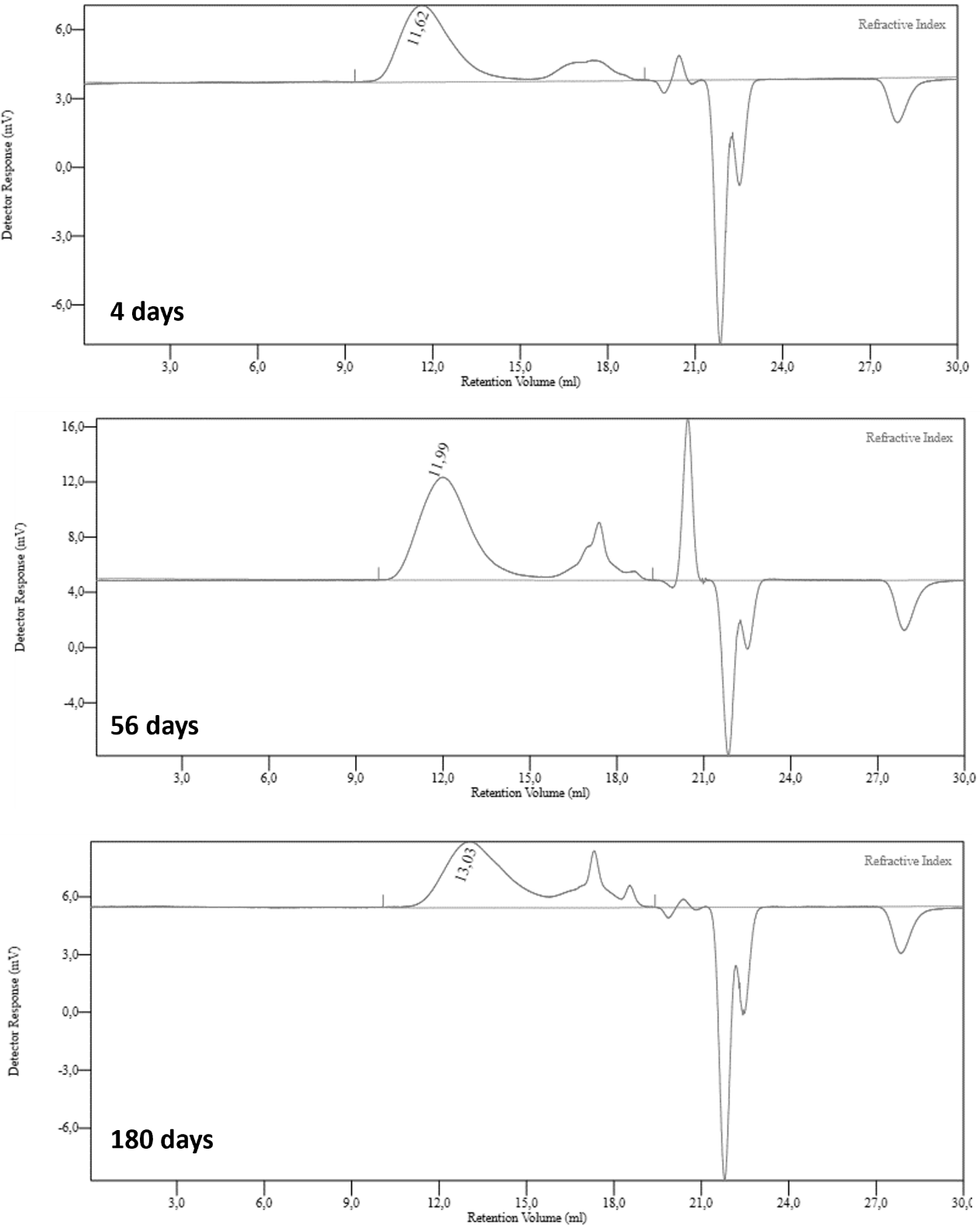
**Figure S9.** Representative evolution of GPC traces over the time of the in vivo degradation period for PLATMC in salt-particulate leached scaffolds. The curves corresponds to time points 4, 56, and 180 days after explantation of the scaffolds and dissolution in chloroform.


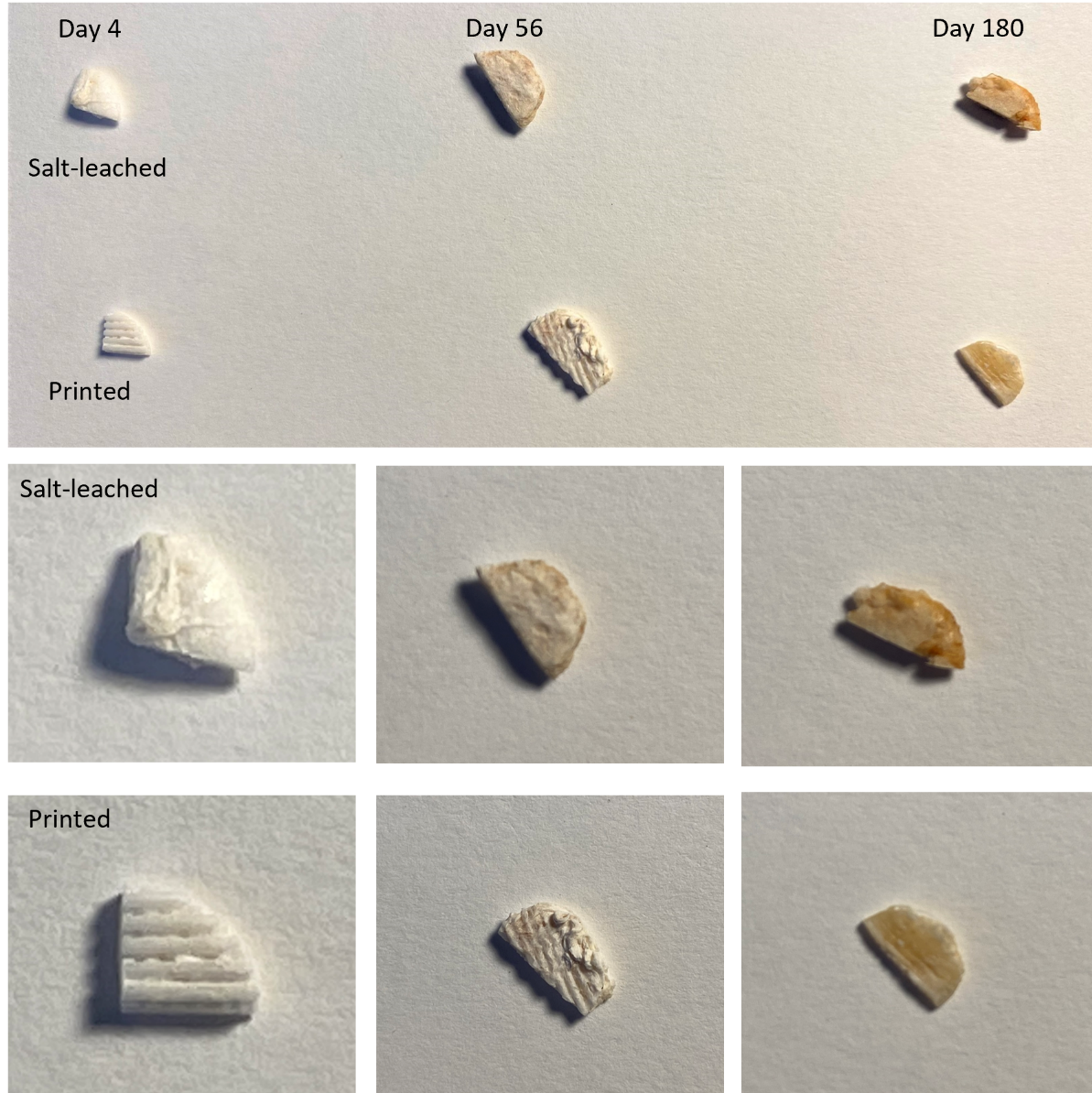
**Figure S10.** Visual representation of PLATMC scaffolds after explantation and cleaning, prior to analysis of key material properties. Top row: salt-leached scaffolds (Days 4, 56, 180). Bottom row: 3D printed scaffolds (Days 4, 56, 180).


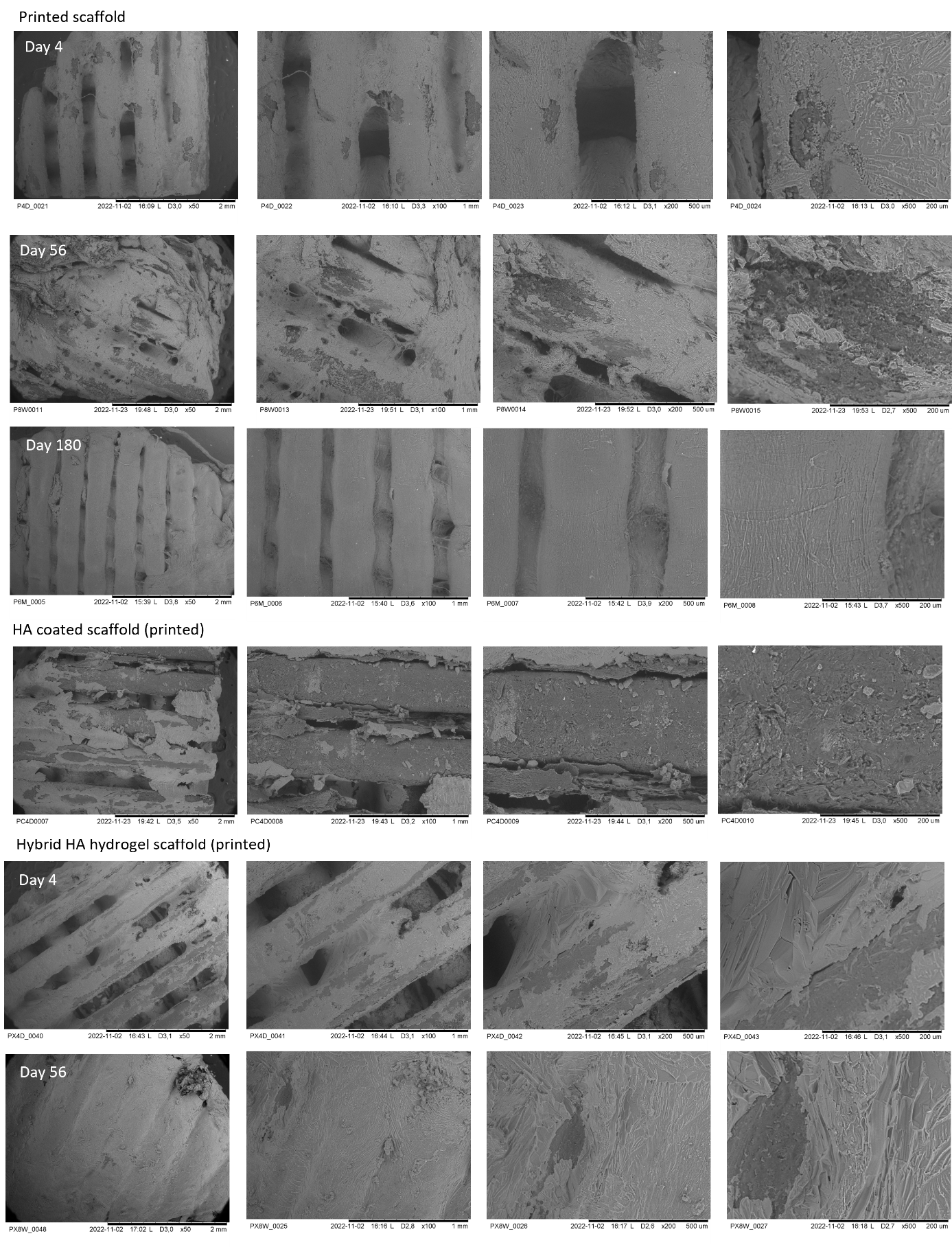
**Figure S11.** SEM images of the printed PLATMC scaffolds during the *in vivo* degradation period. The magnification is x50,x100,x200 and x500.


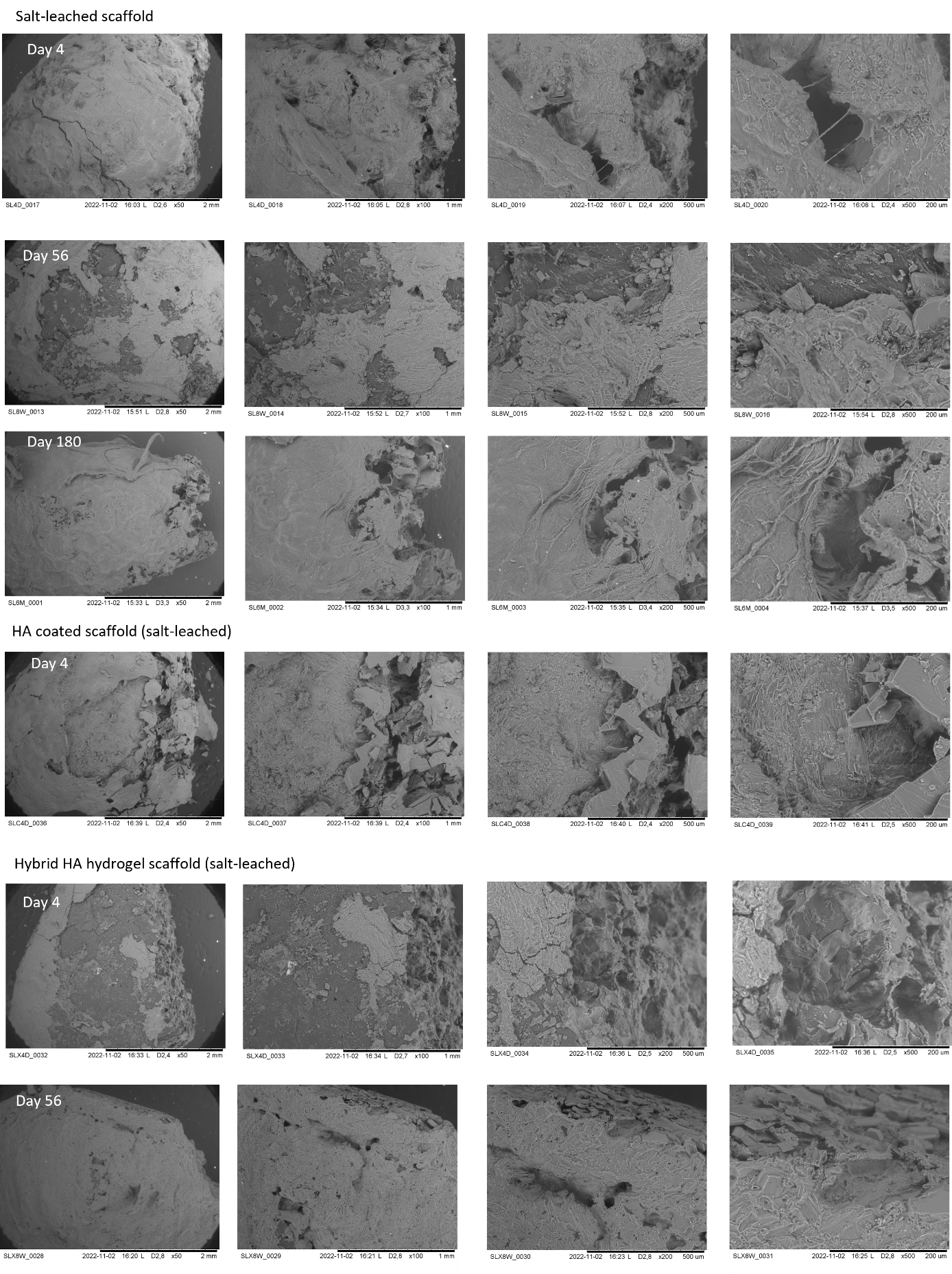
**Figure S12.** SEM images of the printed PLATMC scaffolds during the *in vivo* degradation period. The magnification is x50,x100,x200 and x500.


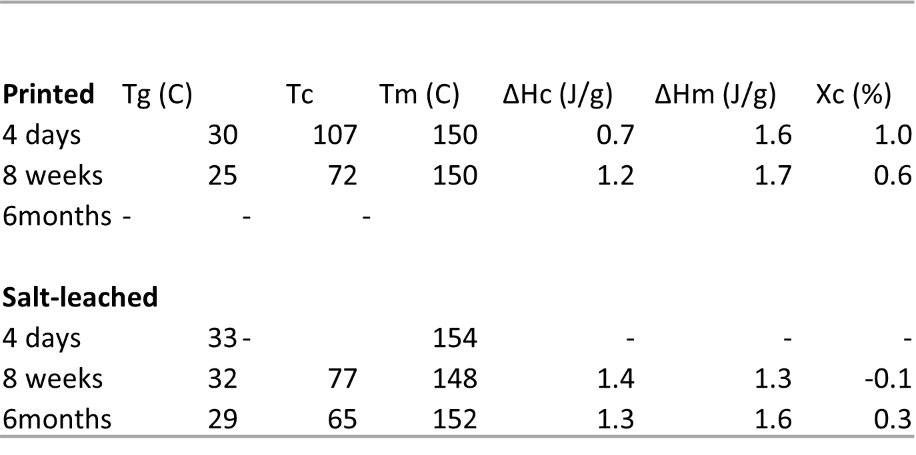

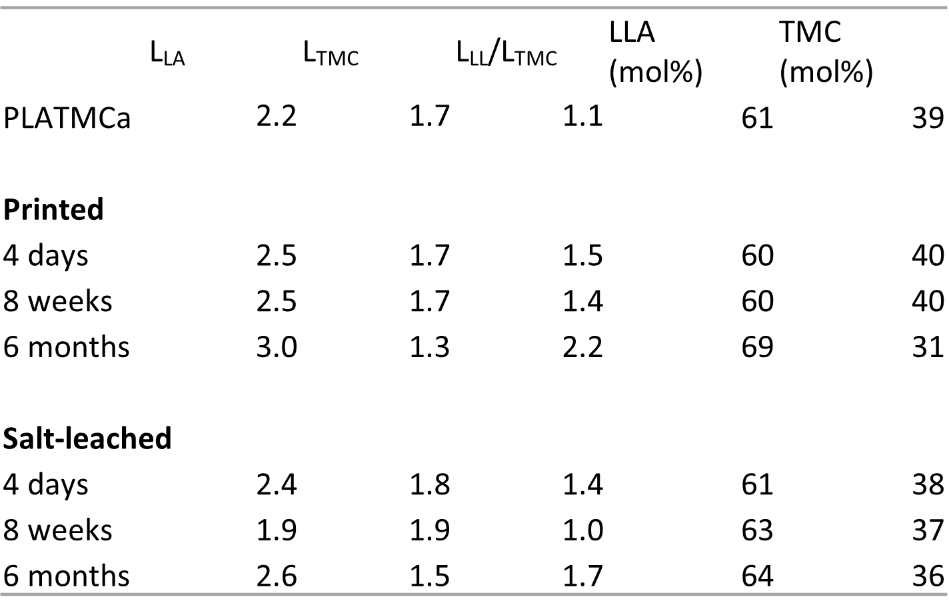
**Figure S13.** Evolution in average chain lenght (LLL and LTMC) and polymer composition (mol% LLA and TMC) over the time of the in vivo degradation period for PLATMC in extrusion based 3D-printed (Printed) and salt-particulate leached (Salt-leached) scaffolds. Values are obtained from 1H and 13C NMR analysis after explantation of the scaffolds on day 4, 56, and 180. a refers to the polymer composition of PLATMC before scaffold fabrication.

**Figure S14:** Thermal properties over the time of the in vivo degradation period for PLATMC in extrusion based 3D-printed (Printed) and salt-particulate leached (Salt-leached) scaffolds. Glass transition temperature (*T_g_*) was derived from midpoint ISO, crystallization point temperature (*T_c_*), melting peak temperature (*T_m_*), enthalpy of fusion (*ΔH_m_*) and cold crystallization (*ΔH_c_*) from the first heating run. Degree of crystallinity (*X_c_*) was calculated assuming *ΔH_m_*°= 93.0 J g^-1^ for 100 % crystalline PLLA. Prior to scaffold fabrication, PLATMC was semi-crystalline with a Tg of 32C a Tm of 158C and the Xc was 20 %.


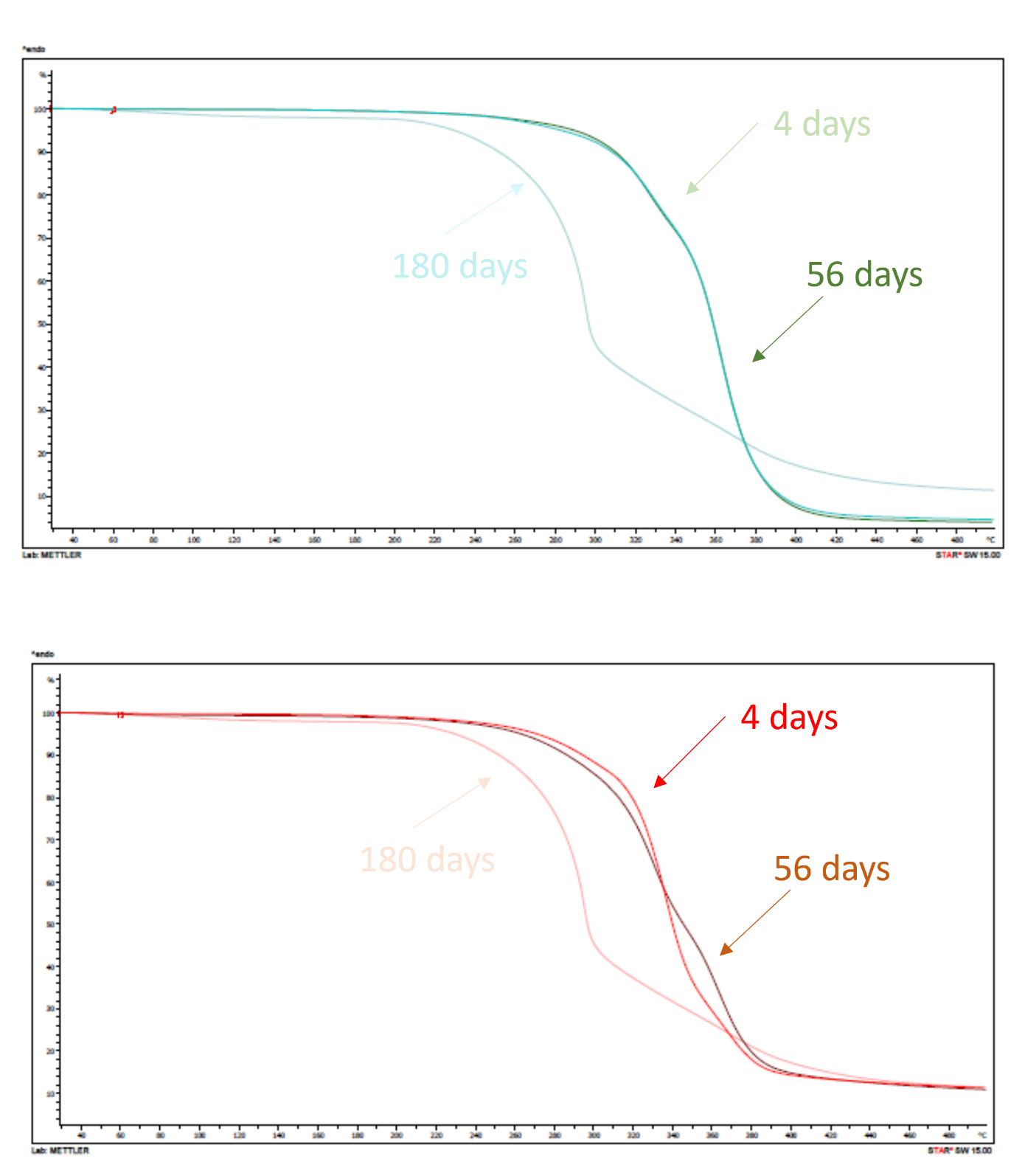
**Figure S15:** Representative weight loss curves of PLATMC from a) printed scaffolds; b) salt-leached scaffolds.


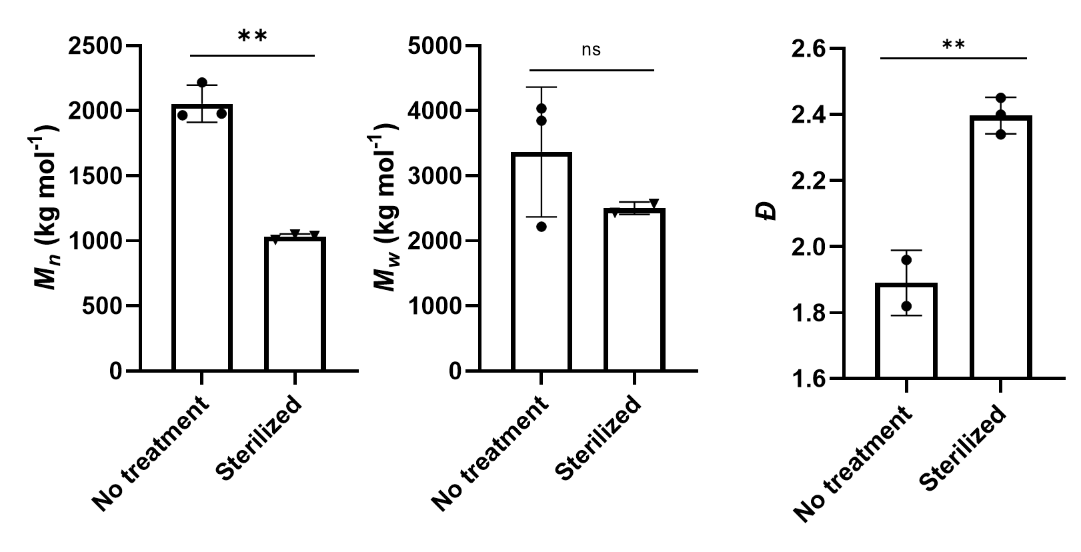
**Figure S16.** Degradation impact on HA during sterilization process illustrates a significant difference in M_n_ and *Đ* of HA after sterilization with ethylene oxide compared to before (no treatment). Based on these data, it should be noted that the scaffolds coated with HA had an M_n_ corresponding to approximately half of the initial M_n_. Determined from Ionex Ultimate-3000 HPLC system referenced to pullulan standards. Statistical significance was determined using Student´s t-test: N.S. = not significant, *p ≤ 0.05, **p ≤ 0.01, ***p ≤ 0.001, ****p ≤ 0.0001 (n= 3; mean ± SD).
